# Microbial Source Tracking of Fecal Indicating Bacteria in Coral Reef Waters, Recreational Waters, and Groundwater of Saipan by Real-Time Quantitative PCR

**DOI:** 10.3389/fmicb.2020.596650

**Published:** 2021-01-18

**Authors:** Christopher Sinigalliano, Kiho Kim, Maribeth Gidley, Kathy Yuknavage, Karen Knee, Dean Palacios, Charito Bautista, Anthony Bonacolta, Hyo Won Lee, Larry Maurin

**Affiliations:** ^1^Atlantic Oceanographic and Meteorological Laboratory, National Oceanic and Atmospheric Administration, Miami, FL, United States; ^2^Department of Environmental Science, American University, Washington, DC, United States; ^3^Cooperative Institute for Marine and Atmospheric Studies, University of Miami, Miami, FL, United States; ^4^Water Quality Surveillance/Nonpoint Source Program, Bureau of Environmental and Coastal Quality, Commonwealth of the Northern Mariana Islands, Saipan, MP, United States

**Keywords:** fecal indicating bacteria, microbial source tracking, land-based sources of pollution, water quality, *Enterococcus*, bacteroides, Saipan

## Abstract

The Commonwealth of the Northern Mariana Islands (CNMI) recently identified the need to improve its capacity for detecting and tracking land-based sources of pollution (LBSP) in coastal waters, particularly microbial contaminants like fecal indicator bacteria (FIB). Reported here is a baseline study of a suite of host-specific FIB microbial source tracking (MST) markers in the coastal shoreline and reef waters around the island of Saipan. Three sampling campaigns were conducted in September 2017, March 2018, and August 2018. Samples were collected from the nearshore surface waters of Saipan, the reef waters of Saipan Lagoon, and groundwater from beaches along the Saipan Lagoon shoreline. Measurements of submarine groundwater discharge (SGD) into nearshore waters and isotopic source tracking of nitrogen inputs were conducted concurrently with MST. Environmental DNA was extracted from the samples and analyzed by quantitative polymerase chain reaction (qPCR) for MST gene markers of fecal Bacteroidales specifically associated with humans, dogs, cows, and pigs, and for an MST gene marker of *Catellicoccus* associated with seabirds. MST assessments were combined with local knowledge, assessments of sanitary infrastructure, and routine watershed surveys. This study identified hotspots of human FIB along the western Saipan Lagoon shoreline in both surface waters and groundwater, plus another hotspot of human FIB at a popular tourist bathing area known as the Grotto. FIB hotspots on the Lagoon shoreline coincided with areas of high SGD and nitrogen isotopic data indicating sewage-derived N inputs. It appears that faulty sanitary infrastructure may be contributing to inputs to Saipan Lagoon, while bather shedding is likely a primary input for the Grotto area. Moderate levels of dog fecal contamination were common and widespread across the island. High levels of seabird fecal contamination were more random, both spatially and temporally, and mostly concentrated along the less developed northeast region of Saipan. No significant levels of cow or pig fecal marker were detected in coastal water samples. This study provides demonstration and establishment of analytical capacity to resource management in CNMI for MST technology to aid in trouble-shooting water quality issues involving land-based sources of microbial contaminants to CNMI coastal waters.

## Introduction

The Commonwealth of the Northern Mariana Islands (CNMI) is a territory of the United States that comprises all the islands of the Northern Mariana archipelago chain in the northwest Pacific, except for the island of Guam (which is its own separate US territory). Saipan, the largest of the 14 islands of the CNMI, is the capital and houses the largest population, with the majority of communities situated along Saipan’s western coast. Waters along this area are negatively impacted by chronic sediment, nutrient, and microbial pollution that, combined with other stressors such as temperature-induced bleaching, ocean acidification, fishing pressures, reef tourism pressures, etc., can affect many of the island’s western and southeastern reefs (Starmer et al., 2008; Yuknavage et al., 2018). Saipan’s geology consists of a volcanic basement overlaid with terraced limestone shelves. Despite abundant rainfall, little fresh surface water exists, and the majority of available freshwater occurs in aquifers due to the high permeability of fragmented limestone. As in other small oceanic islands, most of the available fresh water in Saipan is in a freshwater-saltwater coastal-aquifer system where a lens-shaped body of fresh and brackish ground water floats on denser saltwater within the island (Carruth, 2003). Treated aquifer groundwater provides the primary source of fresh tap water for the island population. Saipan has a variety of potential sources of nutrient, chemical, and/or microbial contaminants to both the groundwater aquifer and surface waters, including groundwater infiltration from septic fields, golf courses, some small-scale agriculture, and small industries, plus point sources to the coastal ocean such as the outfalls from two wastewater treatment plants and brackish effluent from reverse osmosis (RO) water treatment surface impoundments (Carruth, 2003; Perreault, 2007). In addition, there is a recognized issue with elderly and/or failing wastewater infrastructure, leading to elevated levels of fecal indicator bacteria in both beach surface water and groundwater (Knapp et al., 2020). Saipan is bordered by a fringing reef off the western coast with an extensive lagoon system that is hydrodynamically separated into three distinct Saipan Lagoon regions: Tanapag, Garapan, and Chalan Kanoa (Kruger et al., 2010). The Garapan Lagoon region in particular borders the populous middle section of the western coastal plain of the island, with substantial urban development, numerous storm drains, and known challenges of outdated and potentially leaking sewage infrastructure (Yuknavage et al., 2018). Since the 1940s, the Saipan Lagoon system has lost about 20% of its seagrass and coral cover (Perez et al., 2018).

Management of the CNMI’s coastal resources falls under the jurisdiction of the Bureau of Environmental and Coastal Quality (BECQ). The BECQ is responsible for monitoring, assessing, and protecting water quality within the CNMI, as well as managing land, air, water, and coastal quality. Both commonwealth and US federal laws and regulations mandate this responsibility (Yuknavage et al., 2018). As a whole, the CNMI’s marine waters meet the high standards specified by the BECQ for water quality. The majority are designated as “Class AA,” which reflects the highest standard of water quality. However, there are still a variety of point and non-point sources of nutrient and microbial contamination that can impair water quality. These pollution sources have the potential to affect both public and ecosystem health, including coral reefs. Environmental managers from Saipan suspect that surface runoff, stormwater discharges, and submarine groundwater discharge (SGD) may serve as significant conduits of microbial and nitrogen pollution. SGD has recently been quantified in this region, using natural tracers radon (Rn) and radium (Ra) to help identify where SGD is occurring (Knapp et al., 2020).

In recent years, the BECQ has issued numerous water quality violations due to elevated enterococci fecal indicating bacteria (FIB) levels in areas of Saipan with heavy stormwater runoff (Yuknavage et al., 2018). Many of these sites are within the highly developed and urbanized Garapan district along the western coast, where drainage issues are in the process of being addressed. Other frequent violations have occurred within Saipan’s marinas and in waters surrounding docks, as well as at high-density tourist swimming sites such as the Grotto (Yuknavage et al., 2018). A large number of dogs (known locally as “boonie dogs”) also roam the island, especially in developed areas around the central western Saipan Lagoon shore. Dogs can contribute significant loads of fecal bacteria to terrestrial runoff as non-point-source discharges. Dog fecal contamination not only poses a possible public health risk but also potentially confounds water quality assessments based solely on general FIB measurements, as dog feces can contain very high levels of enterococci as compared to some other animals or humans (Solo-Gabriele et al., 2011; Cinquepalmi et al., 2013). A recent population survey of free-roaming dogs in Saipan estimated that more than 21,000 such dogs are on the island (Nimer et al., 2018). Many island residents perceive the large free-roaming dog population as a public health concern, and animal control on the island faces many challenges.

The CNMI has 240.5 miles of ocean shoreline. In 2018, 50.5 of these coastline miles (or 21% of the CNMI’s coastline miles) were found to be impaired due to exceedance violations of enterococci FIB levels (Yuknavage et al., 2018). 32.7 miles of such impaired coastline surrounded the island of Saipan. As in previous years, the BECQ has considered the most common sources of enterococci contamination to be from point sources that include failing sewer lines and other municipal wastewater collection sites, individual onsite wastewater collection systems, and non-point sources. These non-point sources include: (1) sediment-laden storm water runoff with naturally occurring enterococci from urban runoff, erosion from construction sites and new developments, etc.; (2) illicit wastewater discharges from animal pens and outhouses; (3) waste from free-range feral and domestic livestock; and (4) a lack of adequate public restroom facilities at popular, heavily visited tourist sites (Yuknavage et al., 2018).

The BECQ regularly monitors the microbiological water quality of the CNMI’s coastal waters through its water quality surveillance program, testing for enterococci fecal bacteria with the EnteroLert system (IDEXX Laboratories). It also regularly tests for *Escherichia coli* with the ColiLert system (IDEXX Laboratories) as per the manufacturer’s instructions and guidelines by the US Environmental Protection Agency (EPA). However, these current regulatory approved tests lack any source-tracking capability. Although general FIB monitoring is useful for identifying hotspots and zones of water quality exceedance, it is of less utility in helping resource managers understand why there are such exceedances and where the microbial contamination may be originating (Harwood et al., 2014).

The BECQ already uses a GeneDisc Rapid Microbiology System and a commercially available kit (Pall Corporation) to conduct quantitative polymerase chain reaction (qPCR) enumeration of *E. coli* and enterococci FIB. The system consists of a Pall GeneDisc qPCR thermocycler and Pall Environmental DNA Extractor System used with the GeneDisc Recreation Water *E. coli* and *Enterococcus* spp. assay kit (Pall Corporation). However, these particular commercial qPCR assays are for general FIB enumeration and lack source tracking capability. This project has now adapted the in-house BECQ GeneDisc qPCR thermocycler platform for conducting a range of standard host-specific microbial source tracking (MST) assays.

Many strains and species of gut microbial flora have co-evolved with their animal hosts and contain unique gene sequences that can be diagnostic for particular host-associated FIBs, indicating fecal contamination specific to particular host animals such as humans, dogs, birds, pigs, cows, etc. (Boehm et al., 2013). The ability to enumerate the relative abundance of host-specific FIB is a useful tool for resource managers to investigate hotspots of microbial contamination. It also potentially provides better insight into the possible sources and patterns of transport for land-based sources of pollution (LBSP) that contribute to the microbial contamination of impaired waters and enables a better assessment of the potential environmental and public health risks of such contamination (Harwood et al., 2014). Patterns of excessive human-source fecal indicators can suggest infrastructure problems and sanitary leakage, while canine-source FIB markers may indicate increased contamination inputs from surface runoff and stormwater sources. However, it should be recognized that in some urban communities in some countries, there is an increasing tendency for pet owners to flush pet waste into municipal sanitary systems (Caldwell and Levine, 2009). Despite this, flushing dog fecal waste is not believed to be a common practice in Saipan, and most of the dog population on the island is free-roaming outdoors. In addition, agricultural FIB markers such as from cow and pig sources can indicate livestock fecal inputs, while excessive bird markers may indicate background inputs from wildlife populations.

Microbial source tracking adds another set of tools to the resource management toolbox that, in combination with other methods, can better direct investigative and mitigation efforts. MST is a DNA-based technology that enables the water quality management community to determine whether humans or other animal species are responsible for microbial fecal contamination in an aquatic environment. A variety of methods for molecular MST of fecal indicator bacteria have been developed, tested, and deployed, and applications for MST in water quality management are becoming increasingly common (Boehm et al., 2013; Harwood et al., 2014). Note that there are no MST methods promulgated yet for regulatory criteria, and there are currently no abundance threshold exposure limits promulgated in the US for regulatory purposes for any host-specific MST genetic markers. Rather, MST assessment of the relative abundance of host-specific fecal bacterial genetic markers is currently used more commonly in conjunction with the regulatory general FIB assessments for enterococci and *E. coli* as a troubleshooting approach to investigate chronic microbial water quality problems. This type of approach can be highly effective when integrated into a multi-tool, multi-tiered strategy for water quality assessment, as described in The California Microbial Source Identification Manual: A Tiered Approach to Identifying Fecal Pollution Sources to Beache*s* (Griffith et al., 2013) by the Southern California Coastal Water Research Project (SCCWRP).

Bacterial water quality criteria in the CNMI are promulgated by the BECQ and are based upon the EPA’s water quality recommendations (US Environmental Protection Agency [US EPA], 2012b). The current regulatory microbial water quality criteria in the US and its territories rely on the culture-based enumeration of *E. coli* and/or fecal enterococci (Soller et al., 2010a, b, 2014). The recommended regulatory limits for enterococci in recreational waters, measured by either the membrane filtration plate count method as in EPA method 1600 (US Environmental Protection Agency [US EPA], 2009) or the Chromogenic Substrate method as in the commercial EnteroLert test (IDEXX), is a geomean of 35 CFU/100 mL or 35 MPN/100 mL, respectively. In addition to these regulatory water quality criteria, there is also a recommendation for a single-sample Single Threshold Value (STV) for a “Beach Action Value” or BAV limit of 130 CFU or MPN per 100 mL. This value is based upon the 90th percentile confidence interval (CI) for the STV. Note that this BAV is not a regulatory water quality criteria *per se*, but rather serves as guidance toward decision-making about posting bather warnings or beach closure notifications due to bacterial risk, based on the results of single grab samples rather than a geomean of multiple samples over time. Thus, the BAV is the abundance of viable enterococci in recreational waters that would trigger posted beach warnings or beach closures. The EPA equates these values to an estimated illness rate of 36 illnesses per 1000 bathers, based on a series of epidemiological studies (US Environmental Protection Agency [US EPA], 2012b, p. 43). For additional precaution, the EPA has suggested an even lower BAV based on an STV of 70 CFU or MPN per 100 mL to be “more conservative,” based on the 75th percentile CI rather than the 90th percentile CI. The EPA also has an alternative recommendation for an optional higher protective standard consisting of a geomean of 30 CFU or MPN per 100 mL, and a single-sample BAV limit of 60 CFU or MPN per 100 mL. These alternative values equate to an estimated illness rate of only 32 illnesses per 1000 bathers (US Environmental Protection Agency [US EPA], 2012b, p. 44). In regards to enumeration of total enterococci (including non-viable) by qPCR gene amplification, the EPA has recommended a single-sample BAV limit for general enterococci as measured by the Taqman qPCR Entero1A assay as described in EPA method 1611 (US Environmental Protection Agency [US EPA], 2012b), consisting of 1000 “cell calibrator equivalents” (cce) per 100 mL, for a predicted illness rate of 36 illnesses per 1000 bathers, or an optional alternative of 640 cce per 100 mL for a predicted illness rate of 32 illnesses per 1000 bathers (US Environmental Protection Agency [US EPA], 2012b, p. 44). While terminology in the literature may vary, these cce are essentially equivalent to “target genome copies” (TGC), “genome copies” (GC), or “genome equivalents” (GE). It is up to each US state and territory to consider these various EPA recommendations for bacterial recreational water quality standards and then promulgate their own official regulations, water quality criteria, and state/territory specific BAVs, and to provide justification to the US EPA for these state/territory criteria. In the case of the marine Pacific territories of American Samoa, Guam, and the CNMI, the official BAV for the live enterococci single sample threshold is 130 CFU or MPN per 100 mL of water sample based on the 90% CI of the STV, reflective of 36 illnesses per 1000 bathers. The CNMI has not yet officially promulgated a BAV for qPCR-based enterococci abundance.

To our knowledge, there are currently no regulatory criteria, guidelines, or official recommendations for exposure limits or BAVs of any host-source-specific FIB microbial source tracking markers. However, a quantitative microbial risk assessment (QMRA) study by Boehm et al. (2018) focused on the potential exposure risk of the human-source fecal *Bacteroides* qPCR marker HF183 (the Minor-Grove-Binder Taqman version, as in EPA Method 1696). These researchers concluded that the risk associated with exposure to a fixed HF183 concentration in recreational water decreased with the age of contamination, and that swimmer exposure to sewage after it had aged ∼3 days resulted in a median risk of less than 30 illnesses per 1000 bathers. This QMRA study calculated a risk-based HF183 threshold of 900 copies/100 mL for sewage contamination aged 2.5 days, while a risk-based water quality threshold for HF183 in surface waters that takes into account uncertainty in contamination age was derived to be 4100 copies/100 mL (Boehm et al., 2018). Considering this previous QMRA study by Boehm et al., we suggested to the BECQ that when interpreting the results of this Saipan MST study, they utilize an HF183 marker exposure threshold of 1000 copies/100 ml for human-source fecal contamination less than 3 days old and 4100 copies/1000 mL of HF183 marker for human-source fecal contamination of uncertain age or greater than 3 days old.

As part of the broader MST technology transfer project between the NOAA-Atlantic Oceanographic and Meteorological Laboratory (AOML) and the CNMI-BECQ, an initial study was conducted as a collaborative effort between the BECQ, NOAA-AOML, and American University. This study examined land-based sources of microbial pollution to the coastal waters of Saipan following the principles and protocols of the California Microbial Source Identification Manual (Griffith et al., 2013). The goals were to enumerate host-specific FIB markers, targeting fecal contamination from humans, dogs, seabirds, cows, and pigs in nearshore coastal waters, recreational beach waters, and reef-associated surface waters, as well as in selected groundwater samples that discharge into Saipan Lagoon. These data were supplemented with nitrogen stable isotope analyses of nearshore macroalgae and a survey of SGD on Saipan’s western coast to better understand pathways of inputs of land-based pollutants. This information was used for follow-up management recommendations to the BECQ to better inform their resource management efforts regarding microbial and nutrient water quality. An additional goal was to establish an ongoing in-house capability for routine MST analyses in the BECQ laboratory using the qPCR thermocycler instrumentation already in their possession. The process of the MST technology transition to the BECQ is described in more detail in the online Supplementary Materials document of this article, and is also reported elsewhere in detail (NOAA-Coral Reef Conservation Program, Final Project Report for CRCP Project # 31184). In addition, the details of the multi-laboratory validation of GeneDisc performance with MST assays and multi-platform comparisons of performance between GeneDisc and other thermocyclers are intended to be published elsewhere. This report focuses on the results of the baseline MST field study in Saipan’s coastal waters conducted as part of this technology transfer project.

## Materials and Methods

### Study Region and Field Program

Our study area was the island of Saipan, bounded by north latitude 15.290533°, south latitude 15.091500°, west longitude 145.685494°, and east longitude 145.830316°. Sampling sites included Saipan Lagoon and the Saipan reef tract along the western side of the island, sites at the northern end of the island, particularly the Grotto area (site NEB01), Bird Island (site NEB02), selected beaches along the northeast portion of the island, and the Lao Lao Bay area on the eastern side of Saipan.

Field sampling efforts for the Saipan MST baseline study were conducted on March 12-19, 2018 and July 31-August 8, 2018. A few samples of opportunity were collected on September 13, 2017 (before the baseline MST survey started) as part of a routine BECQ monitoring effort, and were used primarily for demonstration purposes to teach the MST qPCR methods to BECQ personnel during the first MST technology transfer workshop conducted by NOAA-AOML. During the actual MST baseline surveys in 2018, BECQ researchers collected almost half of the samples during their routine water quality surveillance efforts, while researchers at American University collected shore and reef samples as a courtesy during their own ongoing nitrogen isotope and SGD tracking studies (Knapp et al., 2020), which occurred simultaneously with our MST studies.

**FIGURE 1:**
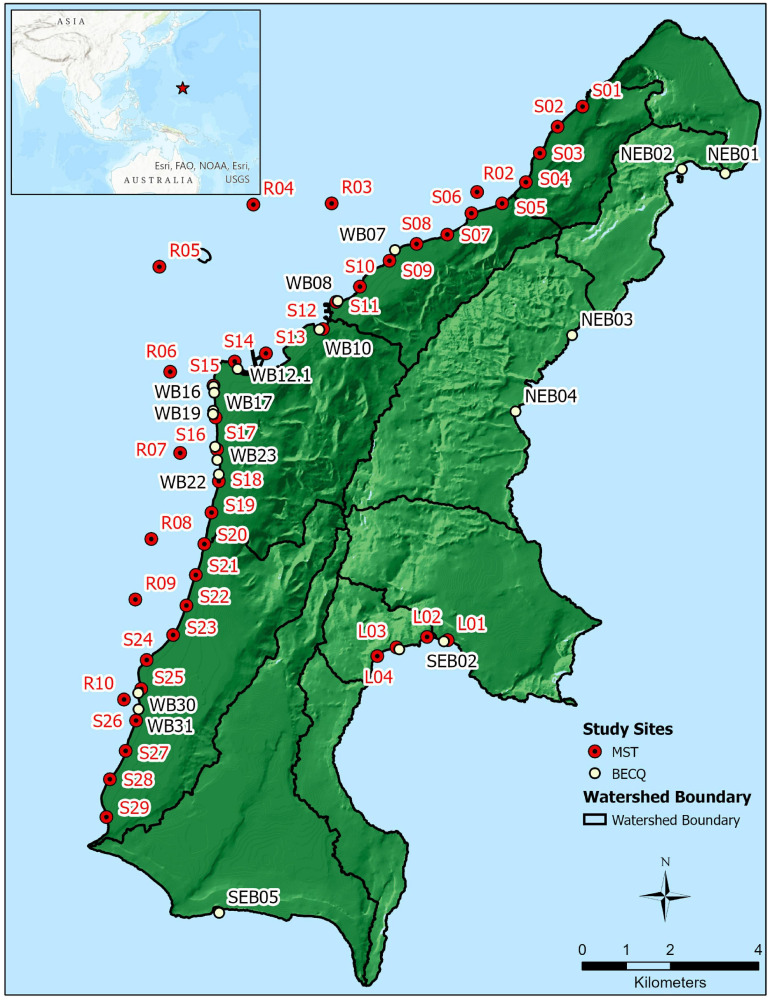
Location of sample sites on the island of Saipan used for MST analyses, plus boundaries of the major watersheds on the island. NEB, SEB, and WB samples were collected by the BECQ from their routine water quality monitoring sites, while S, R, and L samples were collected by American University at additional sites specific for this study. Sites S1–S14 represent the Tanapag lagoon region (designated as “TANA”), sites S15–S25 represent the Garapan lagoon region (designated here as “GARA”), and sites S26–S29 represent the Chalan Kanoa lagoon region (designated as “CHAL”).

Sample sites are shown in Figure 1, with samples collected by the BECQ denoted by site numbers starting with NEB (northeast beach), SEB (southeast beach), and WB (west beach). These sites are part of the BECQ’s regular water surveillance monitoring program. Sites with numbers starting with S (shore), R (reef), and L (Lao Lao Bay) were collected by American University. Groundwater samples correspond to approximately 1 m inland of the same location as the same numbered shore site (for example, S18 and GW-S18 are shore water and groundwater respectively, with GW-S18 at one meter inland of S18). GPS coordinates for samples collected by the BECQ are reported in Supplementary Table 1, and GPS coordinates for samples collected by American University are reported in Supplementary Table 2.

### Sample Collection and Handling

Surface water samples were collected approximately 15–30 cm below the surface in sterile 1.5 L Whirl-Pak bags (Nasco). Samples from these nearshore and reef locations were collected as per EPA methods 1611 and 1696, stored on ice, and filtered within 6 h of collection in the BECQ laboratory at Saipan (US Environmental Protection Agency [US EPA], 2012a; US Environmental Protection Agency [US EPA], 2019). Groundwater samples were collected using a 24′′ (60 cm) PushPoint field investigation porewater sampler connected to a syringe with tubing (MHE Products, East Tawas, MI, United States). Groundwater samples intended for MST qPCR analyses were placed in sterile 1.5 L Whirl-Pak bags and processed in the BECQ laboratory. More specific details about the methods for the groundwater sampling for this study are presented in a separate publication describing nitrogen stable isotope source tracking and SGD quantification with Rn and Ra isotopes (Knapp et al., 2020).

### Viable Enterococci Enumeration via IDEXX EnteroLert

**FIGURE 2:**
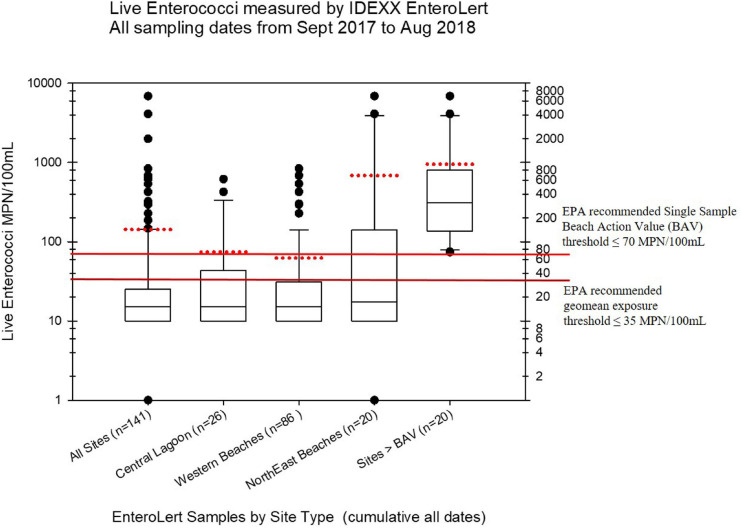
Boxplots summarizing the distribution of live enterococci in the Saipan MST study samples as measured by the regulatory Chromogenic Substrate Most Probable Number Method (i.e., the commercial EnteroLert test by IDEXX Corporation). The black line within the box marks the median, and the red dotted line for each boxplot marks the mean. Note that not all samples were tested by EnteroLert (e.g., only 141 of 182 water samples were tested). The plots show the distribution of enterococci by different regions of Saipan that had hotspots of enterococci exceedances, while the last boxplot shows the distribution of all samples from all sites that exceeded water quality criteria for live enterococci (i.e., exceeding the Beach Action Value with more than 70 MPN/100 mL for a single sample). Fourteen of the 92 samples assessed (15.2%) exceeded regulatory water quality criteria for viable enterococci.

Live enterococci were measured from subsamples of the collected water samples using the standard EPA approved EnteroLert Fluorogenic Substrate method (IDEXX Laboratories), following EPA guidelines, established BECQ protocols, and the manufacturer’s instructions. Samples were processed with the IDEXX EnteroLert method by the BECQ Environmental Surveillance Laboratory. However, not all of samples collected by the American University group were tested using EnteroLert. The viable enterococci abundance in units of Most Probable Number (MPN) per 100 mL as estimated by the EnteroLert assay from the manufacturer’s statistical tables, is shown in Figure 2, and also in the Supplementary Table 5.

### Sample Filtration and Environmental DNA Extraction

In brief, at the BECQ laboratory, aliquots of water samples were filtered through sterile 0.45 μm pore-size, 47 mm diameter membrane filters using disposable sterile filter funnels (Pall Microfunnel, Pall Corporation). Water samples were filtered to 1 L or until clogging, and the volumes filtered varied from <100 mL to 1 l, depending on turbidity (and in the case of groundwater, on how much sample was originally collected). Most samples ranged from 800 mL to 1 L. In all cases, the actual sample volume that was filtered for each individual sample was recorded and used for quantitative calculations of MST target signal. Actual volumes filtered for each sample are listed in online Supplementary Table 5. For the initial samples of opportunity processed in September 2017 as part of the first training demonstrations at BECQ, sterile 0.4 μm pore-size, 47 mm diameter polycarbonate filters were used since these filters came pre-loaded as part of the Pall disposable MicroFunnels (Pall Corporation). For the regular 2018 baseline MST survey samples, cellulose ester filters (Pall GN6, Pall Corporation) were used, so as to be consistent with numerous previous MST studies conducted by NOAA in the marine environment (Sinigalliano et al., 2010, 2019; Campbell et al., 2015; Symonds et al., 2016; Staley et al., 2017). Sample filters were aseptically transferred to Lysing Matrix E bead beat tubes (from the FastDNA Spin Kit for Soil, MP Biomedicals), and preserved by addition of DNAgard Tissue preservative solution (Biomatrica). The MST sample filters were later extracted by gently removing the DNAgard preservative solution, lysing and homogenizing the cells with a SuperFastPrep-2 bead beating instrument (MPBiomedicals), and then purifying total environmental DNA from the lysate with the FastDNA Spin Kit for Soil protocol (MP Biomedicals, Thermo-Fisher) according to the manufacturer’s instructions with minor modifications as described in the online Supplementary Material of this article.

### Modification of MST qPCR Assays for GeneDisc Platform

More specific details of adapting these MST assays to the Pall GeneDisc system are described in the online Supplementary Material to this article. The BECQ laboratory was already in possession of a GeneDisc Rapid Microbiology qPCR System (Pall Corporation), which is an automated user-friendly system that normally requires the use of commercial kits with pre-loaded assay reagents. The GeneDisc system did not have any MST specific assays available at the time of this study, and normally the system cannot be customized by the end user. However, the Pall Corporation graciously custom modified the software of the particular GeneDisc instrument at the BECQ lab and specifically provided our study with custom blank “open” MST GeneDisc plates without pre-loaded reagents. This allowed BECQ personnel to run their own thermocycling parameters and conditions, as well as load their own MST reaction cocktails as per the standard MST protocols in the appendices of the California Microbial Source Identification Manual (Griffith et al., 2013).

The specific MST assays used for this Saipan microbial MST baseline study included: (1) human-source *Bacteroides* assay HF183 (EPA Taqman version as per EPA method 1696); (2) dog-source Bacteroidales assay DogBact; (3) cow-source Bacteroidales assay CowM2; (4) pig-source Bacteroidales assay Pig2Bact; and (5) the Gull2 assay specific for *Catellicoccus marimammalium*. *C. marimammalium* are found in the gut of most seagulls, as well as potentially in the gut of other birds (especially seabirds) that may co-habit, scavenge, or nest with seagulls. Depending on the specific geographic location and co-nesting behavior, this may also include species of terns, pelicans, geese, and very often, pigeons (Sinigalliano et al., 2013). Assay protocols followed the California Microbial Source Identification Manual (Griffith et al., 2013), with minor modifications for the GeneDisc platform (see the online Supplementary Methods Information). The sequences of the oligonucleotides for primers and probes, as well as the synthetic dsDNA standard control fragments, are listed in Supplementary Table 3.

### Quantitative PCR Quality Assurance and Controls

More specific details are given in the online Supplementary Material document. MST qPCR assay quality assurance procedures and controls were as described in EPA Method 1696 for the characterization of human fecal pollution in water by HF183/BacR287 Taqman qPCR (US Environmental Protection Agency [US EPA], 2019). These QA/QC metrics were used as guidance for the QA/QC assessment of all the assays. The standard curve quality control metrics for these qPCR assays as run on the BECQ lab’s Pall GeneDisc instrument in Saipan are shown in Supplementary Table 4. The lower limit of quantitation (LLOQ) was determined for the qPCR reactions from the standard curves of each batch run. Samples with a Cq value greater than the reaction LLOQ were categorized as “DNQ” or “detected but not quantifiable.” The overall reaction sensitivity of the LLOQ for this batch of standard curves was taken to be equivalent to 10 target sequence copies (tsc) per GeneDisc reaction well for each of the MST assays used in this study, which gave a calculated environmental detection LLOQ of 50 tsc/100 mL of water sample. Negative qPCR controls consisted of both “no template controls” (NTC) where no target DNA was added to the reaction wells and method blank (MB) controls where sterile water negative control samples were filtered, extracted, and analyzed in the same manner as the environmental water samples. For sample processing controls (SPC), the variability in sample processing efficiency or inhibition was measured for each environmental sample and method blank sample by using a spike preparation consisting of a fixed concentration of salmon DNA as per EPA method 1696 (US Environmental Protection Agency [US EPA], 2019).

Statistical boxplot analyses of the MST result patterns for host-specific fecal bacterial markers were generated with the SigmaPlot software package, version 14 (Systat Software, Inc.). Final MST positive control concentration standard curves and their associated linear regression statistics were also plotted using the SigmaPlot v.14 software package.

### Stable Isotope Analysis

Sampling of benthic seagrass, algae, surface water and groundwater for isotopic analysis are described thoroughly in Knapp et al. (2020). For seagrasses and algae, five replicates for each species were taken, cleaned of foreign materials, air-dried for at least 24 h at 60°C, and ground to a fine powder. The samples were then weighed in either tin or silver capsules (the latter was used if acidification was required to remove inorganic carbon), and then analyzed on an EA-IRMS. Isotope values are reported as δ^15^N relative to atmospheric N_2_ (δ^15^N = [(R_sample_/R_AIR_)-1]^∗^1000). Two in-house standards, acetanilide and caffeine (iACET, δ^15^N = 1.18‰, caffeine δ^15^N = 20.05‰) were used for calibration and the determination of the precision (0.2‰). Although several species of alga were widespread along the coast, none were present at all sites. Thus, we created an integrated primary producer δ^15^N value using correlations between the most common species, *Caulerpa*, with three others to predict δ^15^N values for *Caulerpa* at sites where it was absent.

Water samples were syringe-filtered (0.2 μm) into 50-mL HDPE screw-top bottles and kept in a cooler until they could be frozen at the end of each sampling day. Then, δ^15^N and δ^18^O were determined using the denitrifier method (Sigman et al., 2001; Casciotti et al., 2002) at Hong Kong University. An IRMS (Thermo Delta V Advantage) was used for isotopic analysis. Isotope measurements are reported in delta notation (‰) relative to atmospheric N_2_ for δ^15^N and V-SMOW for δ^18^O.

### Submarine Groundwater Discharge (SGD)

As detailed in Knapp et al. (2020), SGD was estimated using a combination of measurement of the natural groundwater tracers radon (Rn) and radium (Ra). The activities of these isotopes are generally highest in groundwater, intermediate in coastal waters receiving SGD, and lowest in offshore seawater. Briefly, SGD (m^3^ m^–1^ d^–1^) was calculated based on Ra flux (*F*_*Ra*_; dpm d^–1^) and groundwater endmember Ra activity (*Ra*_*gw*_; dpm m^–3^) at three sites; SGD at other sites was estimated based on the assumption that Ra, Rn, and SGD all followed the same spatial distribution along Saipan’s coast. SGD estimates are given as a range (upper and lower) to reflect sampling error and analytical uncertainty propagated through the calculations.

## Results

**FIGURE 3:**
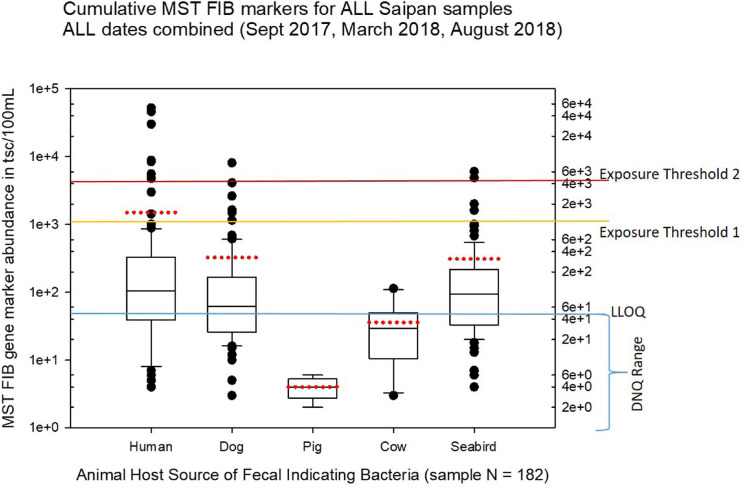
Boxplots summarizing the overall results of MST analyses for host-source fecal indicator bacteria 16S ribosomal gene marker abundance in Saipan coastal waters for all water samples collected during the study from September 2017 through August 2018. The LLOQ line marks the environmental lower limit of quantitation (50 tsc/100 mL). The exposure threshold 1 line marks the suggested HF183 marker exposure limit (1000 tsc/100 mL) for recent human fecal contamination ≤ 3 days, while the exposure threshold 2 line marks the suggested HF183 marker exposure limit (4100 tsc/100 mL) for human fecal contamination of uncertain age > 3 days (Boehm et al., 2018). The boundary of each box closest to zero indicates the 25th percentile, the black line within the box marks the median, and the boundary of the box farthest from zero indicates the 75th percentile. Whiskers (error bars) above and below the box indicate the 90th and 10th percentiles, respectively. The red dotted line for each boxplot marks the mean, and the outliers are shown as dots above or below the whiskers.

**FIGURE 4:**
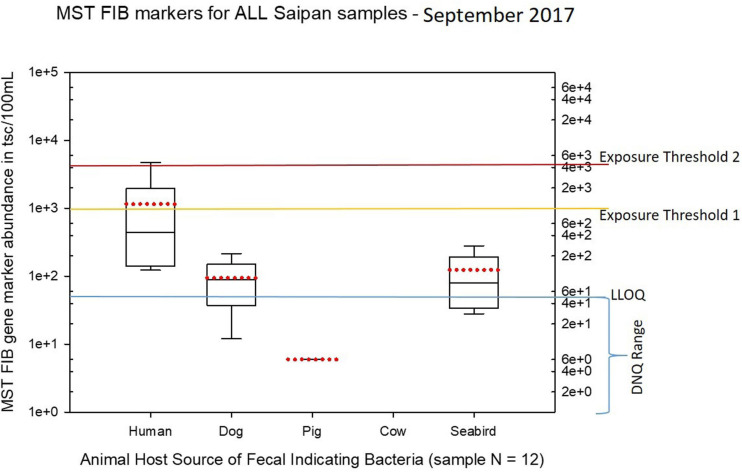
Boxplots summarizing the statistical distribution of the relative abundance of host-specific MST FIB markers for all Saipan coastal water samples collected in September of 2017. Exposure threshold 1 = QMRA suggested limit for HF183 marker from sewage contamination ≤ 3 days. Exposure threshold 2 = QMRA suggested limit for HF183 marker from sewage contamination of uncertain age.

**FIGURE 5:**
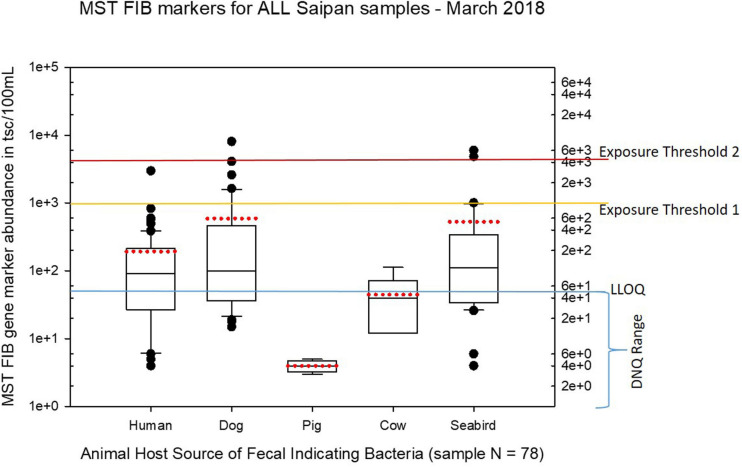
Boxplots summarizing the statistical distribution of the relative abundance of host-specific MST FIB markers for all Saipan coastal water samples collected in March of 2018. Exposure threshold 1 = QMRA suggested limit for HF183 marker from sewage contamination ≤ 3 days. Exposure threshold 2 = QMRA suggested limit for HF183 marker from sewage contamination of uncertain age.

**FIGURE 6:**
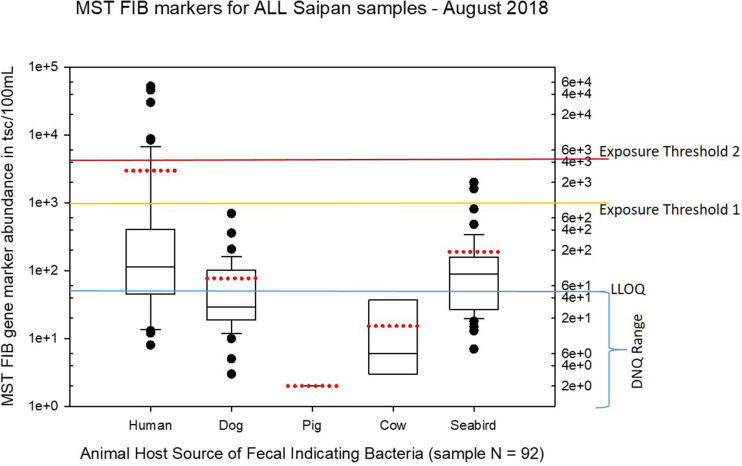
Boxplots summarizing the statistical distribution of the relative abundance of host-specific MST FIB markers for all Saipan coastal water samples collected in August of 2018. Exposure threshold 1 = QMRA suggested limit for HF183 marker from sewage contamination ≤ 3 days. Exposure threshold 2 = QMRA suggested limit for HF183 marker from sewage contamination of uncertain age.

**FIGURE 7:**
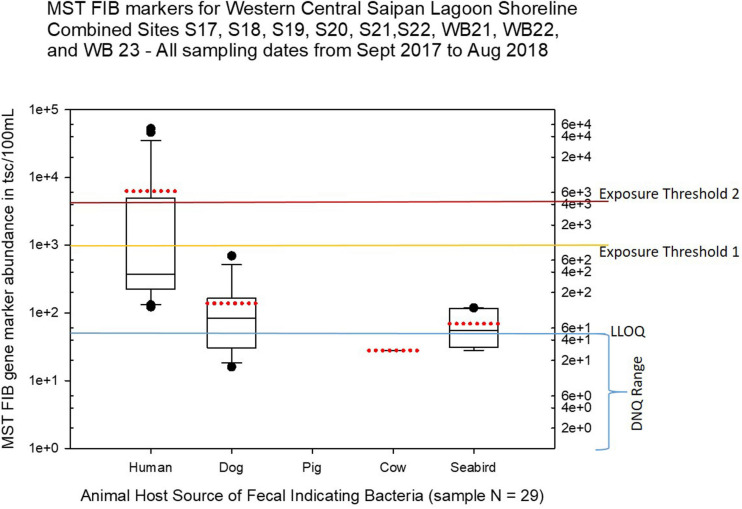
Boxplots summarizing the statistical distribution of the relative abundance of host-specific MST FIB markers for sample sites along the western central Saipan Lagoon shoreline, including sites S17, S18, S19, S20, S22, WB21, WB22, and WB23. Exposure threshold 1 = QMRA suggested limit for HF183 marker from sewage contamination ≤ 3 days. Exposure threshold 2 = QMRA suggested limit for HF183 marker from sewage contamination of uncertain age. All exceedances for QMRA exposure threshold 2 come from sites S18, S20, and S21.

**FIGURE 8:**
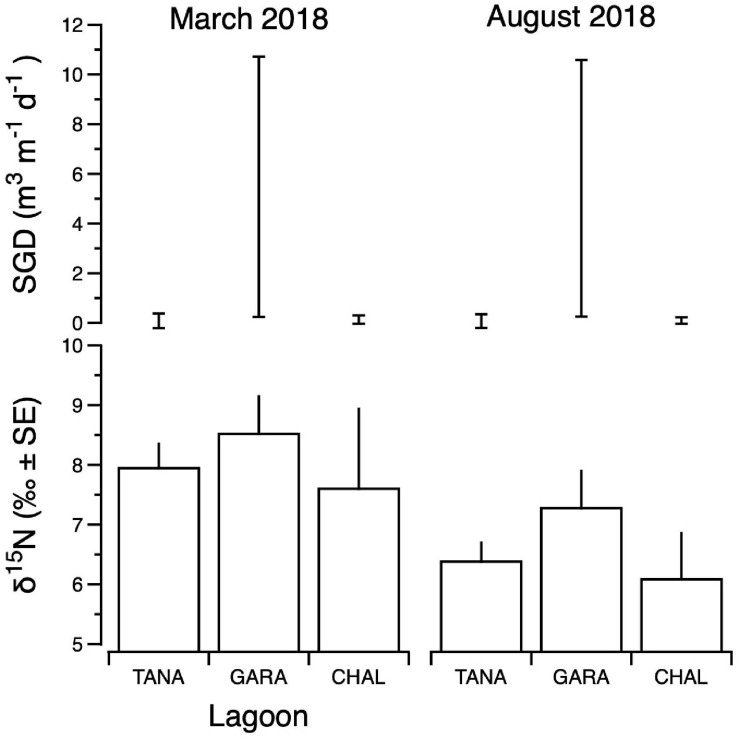
Composite δ^15^N values of benthic primary producers **(lower panels)** and submarine groundwater discharge (SGD) estimates **(upper panels)** for Tanapag (TANA), Garapan (GARA) and Chalan Kanoa (CHAL) Lagoons of the west coast lagoon system of Saipan. For SGD, upper and lower bounds of estimates are shown. Data for two sampling events are shown: March 2018 **(left panels)** and August 2018 **(right panels)**.

**FIGURE 9:**
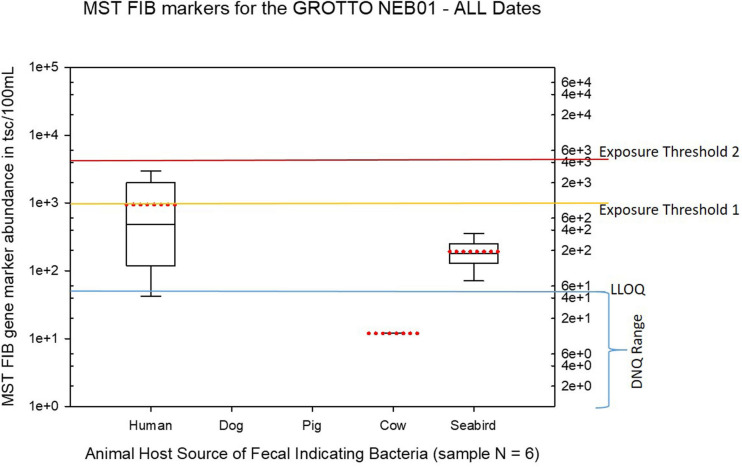
Boxplots summarizing the statistical distribution of the relative abundance of host-specific MST FIB markers for water sample sites collected from the Grotto (site NEB01) for all sample dates from March 2017 to August 2018. Exposure threshold 1 = QMRA suggested limit for HF183 marker from sewage contamination ≤ 3 days. Exposure threshold 2 = QMRA suggested limit for HF183 marker from sewage contamination of uncertain age.

**FIGURE 10:**
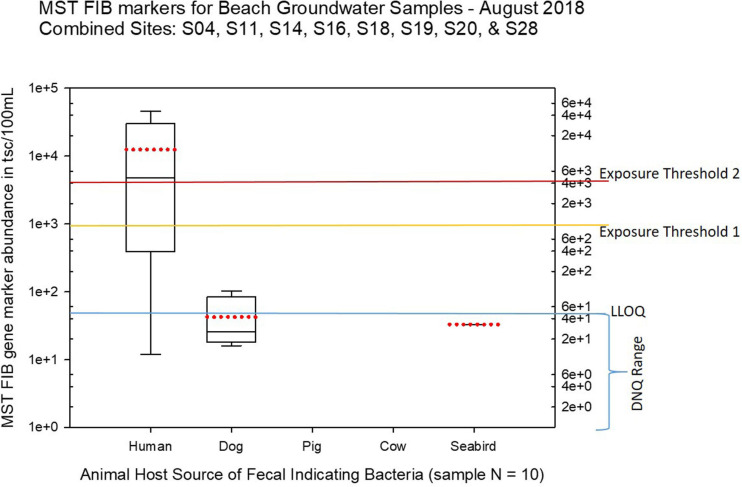
Boxplots summarizing the statistical distribution of the relative abundance of host-specific MST FIB markers for groundwater sample sites along the western central Saipan Lagoon shoreline, including sites S17, S18, S19, S20, S22, WB21, WB22, and WB23. Exposure threshold 1 = QMRA suggested limit for HF183 marker from sewage contamination ≤ 3 days. Exposure threshold 2 = QMRA suggested limit for HF183 marker from sewage contamination of uncertain age. All of the exceedances of the QMRA exposure threshold 2 come from sites S18 and S19.

The summary of results of all fecal indicator bacteria measurements for this study are shown in the project data summary table in the online Supplementary Table 5 of this article. A series of boxplots visualizing the live enterococci measurements by IDEXX EnteroLert Chromogenic Substrate assay are shown in Figure 2, while a series of boxplots visualizing regional MST results are shown in Figures 3–7. Figure 8 depicts submarine groundwater discharge and stable nitrogen isotope observations, while specific site MST results are visualized in Figures 9–10. These boxplots summarize the overall results of the qPCR-based MST for the host-source fecal indicator bacteria gene marker abundance in Saipan coastal waters for water samples collected from September 2017 through August 2018.

The BECQ reported non-detects for IDEXX EnteroLert as <30 MPN/100 mL based on the IDEXX EnteroLert statistical tables for MPN. For the live enterococci boxplots shown in Figure 2, these EnteroLert non-detect values were set at 10 MPN/100 mL for plotting purposes (hence there are no lower whiskers or outliers below the 10 MPN/100 mL level). The lower exposure threshold line shown on these plots represents the 2012 recreational water quality criteria recommendation by the EPA for a geomean of samples (35 MPN/100 mL), for which the EPA has determined these enterococci abundances reflect a predicted illness rate of less than 36 illnesses per 1000 bathers (US Environmental Protection Agency [US EPA], 2012b, p. 43). The upper exposure threshold line represents the EPA recommended BAV for the STV of individual grab samples (70 MPN/100 mL However, it should again be noted that the CNMI BAV as promulgated by the BECQ is actually 130 MPN/100 mL), which is based on the 90% CI reflective of 36 illnesses per 1000 bathers. The US EPA has lowered their suggested BAV to the 75th percentile as it is assumed to be potentially “more conservative” in their suggested protective threshold for posting beach warnings and/or beach closures.

For viable enterococci, 14 of the 92 EnteroLert samples (15.2%) exceeded the EPA recommended BAV threshold of ≤70 MPN/100 mL. The statistical distribution of these exceedances is shown in the last boxplot on the right side of Figure 2. The highest and most frequent enterococci exceedances for these particular samples clustered primarily in the region of the northeastern beaches, including the Grotto (NEB01), Bird Island (NEB02), Jeffrey’s Beach (NEB03), Old Man by the Sea (NEB04), and Hidden Beach (NEB07). Hidden Beach and Old Man by the Sea, which are close to one another geographically in the same watershed, had the highest levels of observed viable enterococci during the study (6,867 MPN/100 mL and 1,989 MPN/100 mL respectively, occurring on August 8, 2020). Enterococci elevations substantially above the BAV were also observed on multiple occasions for Ladder Beach at the southern end of the island (site SEB05). Along the western coast, elevations of viable enterococci above the BAV were observed at the American Memorial Park Drainage (site WB12.1), Garapan Fishing Dock (WB21), Garapan Drainage (WB23), and Saipan Lagoon shore sites S10 and S21.

Figure 3 shows boxplots summarizing the statistical distribution of each MST marker for all cumulative sites and cumulative sampling events for this study, displaying the full range of variation and average abundances of each marker for all the sites and times combined. In Figures 3–10, the calculated environmental LLOQ and DNQ range are indicated in the lower horizontal line marked “LLOQ.” The horizontal line marked “exposure threshold 1” indicates our suggestion to the BECQ for an exposure threshold of 1000 copies/100 mL for the HF183 human *Bacteroides* qPCR marker in surface water for recent human fecal contamination (≤3 days old), while the “exposure threshold 2” line indicates our suggested bather exposure threshold for the HF183 marker in surface water for human fecal contamination of uncertain age or >3 days old. Our suggestions for these HF183 exposure thresholds used to help interpret the results of this Saipan MST study were adapted from information by a previous Quantitative Microbial Risk Assessment study of the HF183 marker (Boehm et al., 2018).

Our study found that both human and dog fecal bacterial genetic markers were relatively widespread in the Saipan surface water sites tested, while the seagull/seabird marker appeared to be somewhat more localized. Both agricultural markers for cow and pig fecal bacteria were much lower than had been anticipated; they were only rarely observed during the study and never above the background DNQ range (i.e., below the environmental LLOQ). As we utilized synthetic DNA gene blocks containing the assay target sequences for quantitation standards, we report our qPCR results here as “target sequence copies” or tsc. While terminology in the current literature varies, this “tsc” unit used here is essentially equivalent to the more common term “gene copies” (GC) or “target copies” (TC) or just “copies” as used in other papers. Note that tsc does not necessarily represent a “genome equivalent” (GE) or “cell calibrator equivalent” (CCE), as genomes may have multiple target copies of a particular assay target gene per genome depending upon the target gene of the qPCR assay (for example the 16S ribosomal targets such as HF183 can have multiple copies per genome, whereas CowM2 is a single copy target per genome).

For the human-source fecal *Bacteroides* HF183 marker, 59 samples out of 182 total samples (32.4%) demonstrated levels over 100 target sequence copies (tsc) per 100 mL, 11 samples out of 182 were over 1000 tsc/100 mL, and 8 samples out of 182 were over 4100 tsc/100 mL. This indicates a 6.0% exceedance rate for the suggested HF183 exposure threshold for recent human fecal contamination in surface waters and a 4.4% exceedance rate for the suggested exposure threshold for fecal contamination of uncertain age. These exceedances, primarily clustered along the shoreline of the western central Saipan Lagoon region, ranged from sites WB17 to S22, plus site NEB01 (the Grotto).

Seasonal differences in the distribution of MST FIB markers for the three sampling campaigns are shown in the boxplots of Figures 4–6 for September 2017 (Figure 4), March 2018 (Figure 5), and August 2018 (Figure 6). The September 2017 data set had two observed exceedances of the suggested QMRA exposure limit for the HF183 marker of 1000 tsc/100 mL: these were the Grotto (site NEB01) at 1,012 tsc/100 mL and the Garapan Drainage #1 (site WB17) at 4,786 tsc/100 mL. In March 2018, there was only one exceedance of the QMRA suggested HF183 exposure threshold of 1000 tsc/100 mL at the Grotto (site NEB01) with an observed level of 3,003 tsc/100 mL on March 16, 2018. However, during the August 2018 sampling event, there were four HF183 exceedances of the exposure threshold 1 of 1000 tsc/100 mL for surface water samples, with all exceedance sites clustered in the western central Saipan Lagoon shoreline area: Garapan Fishing Dock (site WB21) at 1,430 tsc/100 mL, site S18 at 8,442 tsc/100 mL, site S20 at 52,410 tsc/100 mL, and site S21 at 8,819 tsc/100 mL. This same western central Saipan Lagoon shoreline area in the Garapan Lagoon region also showed high elevations of HF183 human *Bacteroides* FIB marker in beach groundwater samples. Groundwater site GW-S18 had 46,062 tsc/100 mL on August 3 and 30,293 tsc/100 mL on August 7, while groundwater site GW-S19 had 5,550 tsc/100 mL on August 3 and 4,814 tsc/100 mL on August 7 (see Supplementary Table 5 for more details). It should be noted that these extreme levels of the HF183 marker over the QMRA suggested threshold of 4100 copies/100 mL were relatively close to one another (American University sample sites S18, S20, and S21) and maintained this elevated level over several days in the region of the western central Saipan Lagoon shoreline. Boxplots showing the statistical pattern of MST markers, particularly for the western central Saipan Lagoon shoreline region, are shown in Figure 7. The pattern for submarine groundwater discharge and nitrogen stable isotope tracking is shown in the boxplots for Figure 8, and the patterns for MST markers in groundwater specifically for the Garapan Lagoon region is shown in the boxplots of Figure 9. In this particular area of the Saipan Lagoon, human fecal marker input was predominant in both frequency and abundance as compared to the other fecal host markers tested.

Integrated primary producer δ^15^N values ranged from 4.3‰ (Tanapag region, August) to 12.0‰ (Garapan Lagoon region, March) and were generally higher in samples from the Garapan region of the Lagoon, and among those samples collected in March (Figure 8). SGD was also variable, ranging from a low of 0.01 m^3^ m^–1^ d^–1^ (Tanapag Lagoon, March) to 10.9 m^3^ m^–1^ d^–1^ (Garapan Lagoon, March) and overall, was most prevalent in Garapan Lagoon (Figure 8). Overall, the data pointed to an area of consistent SGD-borne, sewage-derived N inputs in Garapan Lagoon (sites S17–S21).

The Grotto (Site NEB01) frequently had elevations of both live enterococci and human fecal HF183 marker, plus some elevations of seabird marker, as shown in Figures 2, 10. For the Grotto water samples in Figure 10, several HF183 exceedances of the suggested threshold of 1000 copies/100 mL were observed in multiple samples, but HF183 exceedances were not observed for the exposure threshold of 4100 copies/100 mL.

## Discussion

As part of the broader NOAA technology transition effort to aid BECQ in developing an in-house capacity for qPCR-based molecular MST within the Bureau for Environmental and Coastal Quality, NOAA-AOML and CNMI-BECQ researchers conducted a joint MST baseline study to assess the patterns of host-source FIB from LBSP in the coastal waters of Saipan. This study coincided with and was supported by sampling assistance from American University, which was conducting a separately funded independent study in Saipan of nutrient measurements, nitrogen stable isotope source tracking for nutrient pollution, and tracking radon (Rn) and radium (Ra) isotopes as natural tracers of SGD (Knapp et al., 2020).

Our study found that the predominant inputs of FIB into the critical and protected Saipan Lagoon system and reef tract ecosystems were primarily from human and dog fecal sources. Birds were also a major contributor of fecal bacteria at certain sites, especially along the more sparsely settled northern and northeastern parts of Saipan. The distribution of dog fecal bacteria contamination was relatively ubiquitous around the island and was especially prevalent in the Tanapag area, the urbanized Garapan district, and along the central western coastline bordering Saipan Lagoon. In addition, this Garapan Lagoon region of the Saipan Lagoon system is subject to substantial submarine groundwater discharge (SGD), especially in the sections from sites S17 to S21, which also carry sewage-derived N into the coastal environment (Figure 8).

Two primary hotspots with high levels of human FIB were observed: a wide stretch of the urbanized and developed central western Saipan Lagoon shoreline in the Garapan Lagoon region, and also at the Grotto, an isolated coastal grotto swimming and scuba diving area on the northeast end of the island that attracts extremely large numbers of tourists. Both areas have also been noted as chronic hotspots for elevated enterococci during routine water quality monitoring by the BECQ, although there are other chronic enterococci hotspots previously observed by the BECQ around the island where we did not observe associated high levels of the human marker during this study.

For our study, it was assumed that human fecal contamination, particularly from municipal sewage with a large population of contributors, was likely to be the highest risk fecal input source for water contamination (Soller et al., 2010a, b, 2014). One could cautiously speculate that the other fecal bacterial MST markers used in this Saipan study might be assumed to have similar exposure thresholds of at least the same scope (or more likely even higher thresholds) to that of that human fecal marker, for a similar level of relative public health risk, since the animal fecal sources might potentially be less risky than the human sources. If the more protective human HF183 exposure threshold (i.e., sewage < 3 day old) level of 1000 copies/100 mL is accepted for a predicted illness rate of less than 30 illnesses per 1000 bathers, then one might also apply this same threshold to the interpretation of the other MST markers used in this study, and be reasonably confident of being conservatively protective of public health. However, it must be recognized that bacteria targeted by different fecal source markers will have different attenuation/die-off rates to the *Bacteroides* targeted by the HF183 marker, and some experiments have shown that the source of feces can affect die-off of fecal bacteria and the environmental persistence of fecal source tracking markers (Korajkic et al., 2013, 2019). We thus suggested that for purposes of interpreting MST results from this Saipan study, levels below this value of 1000 copies per 100 mL probably would not reflect a significant public health concern for any of the MST markers used in this study. Therefore, given a lack of epidemiological or QMRA data on the relationships of illness and other host-specific MST markers, we applied the same HF183 risk exposure guidelines as adapted from Boehm et al. (2018) to the other host-source MST markers used in this study. We believe this should be conservatively protective given the likely relative risk of the different fecal host sources.

Although there is a moderate degree of cattle ranching and pig farming on the island of Saipan, the relative levels of both cow and pig fecal Bacteroidales markers in the coastal waters tested were observed to be quite low, with the majority of samples being either non-detects or in the DNQ range. No samples exceeded the 1000 copies/100 mL exposure threshold for either cow or pig fecal Bacteroidales marker. The reason for the lower than expected detection of these agricultural markers is unclear. Previous studies have observed that dietary changes and the age of cattle can influence the abundance and distribution patterns of bacterial fecal source tracking markers in cattle, including both *Bacteroides* and *Enterococcus* species of fecal bacteria, as well as fecal pathogens (Busato et al., 1999; Shanks et al., 2010, 2014; Raith et al., 2013). It is possible that seawater dilution factors, as well as attenuation of marker signal with the distance traveled (via land runoff, stormwater discharge, etc.), might have been a factor in the lower detection of the agricultural fecal markers in coastal waters, especially if the original signal was relatively low in abundance. Also, the particular CowM2 marker used here specifically targets a bovine-host Bacteroidales single-copy gene sequence predicted to encoded for membrane-associated and secreted proteins (Shanks et al., 2006, 2008). Many host-specific MST qPCR assays target ribosomal genes, which are generally considered highly desirable genetic targets as they are highly conserved and each bacterial genome may contain many copies of the ribosomal gene target, making it easier to detect a small number of cells at low concentrations. However, the development of bovine-specific qPCR assays has been restricted in part by the limited amount of genetic variation in the rRNA genes of bovine-associated *Bacteroidales* (Shanks et al., 2008). The currently available host-specific qPCR methods targeting ribosomal genes designed to detect bovine fecal pollution can typically discriminate only between ruminant and non-ruminant sources (Layton et al., 2006; Reischer et al., 2006). The CowM2 target used here, putatively a single-copy gene target, exhibits high levels of host specificity for bovine-associated *Bacteroidales*, and high reaction sensitivity, but it may not be evenly distributed across cattle populations, and as a single-copy target it may have a substantially lower environmental detection sensitivity than the more general multi-copy ribosomal ruminant gene markers (Shanks et al., 2008). This might make the CowM2 marker more susceptible to target signal attenuation, dilution, and decay during transport to coastal waters. In addition, while the CowM2 and Pig2Bact markers have shown high reaction sensitivity and specificity in many studies in North America, Europe, and Australia, they have not always shown similar sensitivity in other geographic areas with cattle populations, such as in India (Odagiri et al., 2015). It might be that dietary or population characteristics of the particular livestock herds in Saipan may be affecting the sensitivity of these agricultural fecal marker assays. This is the first time these MST assays have been used for any livestock of the Mariana Islands archipelago. Therefore, it is likely that further optimization for detection sensitivity testing of the cow and pig markers for the particular local Saipan livestock populations may be warranted, as well as conducting qPCR testing for broader multi-copy ribosomal ruminant markers, such as the Rum2Bact assay (Mieszkin et al., 2010). We have recommended that BECQ follow up with future studies incorporating both a Saipan-optimized CowM2 assay and the more general Rum2Bact ruminant marker assay.

In examining Saipan waters, three samples showed the HF183 human FIB marker to be over 10,000 copies per 100 mL (i.e., substantially above the QMRA-based exposure threshold of 4100 copies/100 mL for human fecal contamination of uncertain age). The HF183 levels observed in these samples reflect a potentially serious exposure risk to public health and may be suggestive of significant leakage of sanitary infrastructure at those sites on those sample dates. It should be noted that these extreme exceedances of the HF183 marker occurred at sites relatively close to one another (sites S18 and S20) and repeatedly over the course of a few days in the region of the western central Saipan Lagoon shoreline. Two of these extreme exceedance samples were from groundwater samples at site S18, collected on August 3 and August 7 respectively, in 2018. The third extreme level of human FIB marker was observed in the shoreline sample of site S20 collected on August 2, 2018. S18 was consistently the site with the highest SGD, and S20 was also located within the high-SGD area of Garapan Lagoon. Additionally, SGD across all sites was higher in August (rainy season). This suggests that SGD may transport dangerous levels of human fecal pollution to the coast. All shore sites in this region of the Saipan Lagoon had elevated HF183 marker levels and accounted for the majority of the exceedances of the QMRA exposure thresholds for our data set (with the other primary exceedance site for HF183 being at the Grotto). The urban area of west central Saipan has substantial aging sanitary infrastructure, and it is probable that the observed elevations in human fecal marker were associated with compromised and leaking sanitary infrastructure sources in the area. Indeed, following the conclusion of this study, some compromised sanitary infrastructure in the region of highest human fecal markers (particularly in the groundwater) was later discovered and addressed for mitigation.

This region of the west central Saipan Lagoon shoreline also had elevated dog fecal bacterial marker in many of the samples. Dog fecal bacterial marker appeared to be relatively widespread about the island, with many sample sites having significant elevations, including the Tanapag Meeting Hall area, the GrandVrio and Fiesta hotels, the Central Repair Shop area, the Garapan Fishing Dock area, and throughout the western central region of the Saipan Lagoon shoreline. Dog fecal contamination has been shown to have a substantial impact on microbial water quality (Garfield and Walker, 2008; Wright et al., 2009; Cinquepalmi et al., 2013). Although previous studies have found that gene markers associated with dog feces may be present in some sewage influents, especially in some highly urbanized communities (Caldwell and Levine, 2009), plus the fact that the US EPA, the Humane Society, and other organizations have recommended flushing pet fecal waste as a safe disposal method, the collection and flushing of dog fecal waste in Saipan is not a typical practice. There is a very large outdoors free-roaming dog population (more than 21,000 at last survey in 2018) which is largest in urban areas (Nimer et al., 2018). Also, we did not detect significant levels of dog fecal marker in the ground water samples tested, whereas some groundwater samples had very high levels of human fecal marker (Supplementary Table 5), however we did detect elevated levels of dog marker in surface waters of this area. This is why we are suggesting that the observed dog fecal marker in Saipan coastal waters most likely represented stormwater/runoff transport mechanisms, while human fecal marker most likely represented sewage/septage inputs. In general, there were no effective dog fecal cleanup activities in place at beaches or inland areas, as well as no dog fecal hygiene policies, at the time of this study. As such, dog fecal contamination appears relatively commonplace and widespread in runoff and stormwater discharge, particularly in the more developed parts of the island.

The results of the nitrogen stable isotope nutrient tracking and Rn-derived SGD measurements from the Tanapag, Garapan, and Chalan Kanoa regions of the western Saipan Lagoon are generally consistent with the qPCR MST observations from this area. The δ^15^N and SGD observations from this study have been separately published in more detail elsewhere (Knapp et al., 2020). In summary, three clusters of sites along the western coast were identified — around sites S8, S18, and S26 — that had δ^15^N values greater than 10‰ in seagrasses and algae. These δ^15^N values are consistent with either a sewage or manure N source to these parts of Saipan Lagoon, but sewage is more likely for several reasons. Agricultural practices that use or generate manure are not very prevalent in this area (United States Department of Agriculture [USDA], 2020), but human population centers with failing wastewater infrastructure are. Additionally, elevated δ^15^N in algae was spatially correlated with human fecal HF183 MST markers, which would not originate from manure. In contrast, algae collected along the reef line had comparatively lower δ^15^N values, indicating limited availability of sewage-derived nitrogen offshore. It was further observed that Rn measurements highlighted the spatial and temporal variability in the input of groundwater into Saipan Lagoon. As expected, groundwater inputs were higher during August (i.e., the rainy season) and were the most pronounced near site S18. When surface and groundwater were analyzed for nutrients, groundwater nitrate concentrations were nearly an order of magnitude higher than those in surface water, indicating groundwater flow was an important pathway of nitrogen pollution into Saipan Lagoon at the time of the study.

These observations of nutrients, stable nitrogen isotopes, and the natural SGD tracer Rn were consistent with observations of the patterns of human FIB input into this same area of Saipan Lagoon. Our MST study along with the stable nitrogen isotope and SGD study collectively observed the groundwater and shoreline samples from this area for sites S18–S22 to simultaneously have elevated human-source fecal bacteria, elevated nutrients, and elevated Rn, strongly implicating contaminated groundwater input to the Saipan Lagoon in this area. The greatest chronic export of high levels of FIB, and particularly high-risk human-source FIB appeared to come from groundwater discharge and shoreline runoff discharge along the western central Saipan Lagoon region stretching from roughly the Garapan Fishing Dock to Susupe, with the worst of the discharge in areas near sites S18 through S20. This is likely the LBSP microbial contaminant source causing the greatest risk exposure of LBSP pathogens to the coral reefs of Saipan Lagoon (at least at the time of our study). Reef waters just offshore from this area also showed the greatest abundance of LBSP microbial contaminants, particularly human FIB marker.

The other hotspot for the HF183 human fecal marker was at the Grotto, site NEB01, a popular tourist destination lacking sufficient sanitary infrastructure. There is only one restroom facility near a parking lot at the top of a cliff located a distance from the Grotto. The Grotto is protected from open ocean wave action, but is tidally flushed. It often experiences high densities of swimmers, bathers, scuba divers, and general tourists who frequently visit this natural attraction in large groups. The swimming areas of the Grotto can only be accessed by descending a very long, steep stairway cut into the cliffside. Large crowds often move up and down the stairway, making transit into and out of the Grotto area difficult and time consuming. There are no restroom facilities in the Grotto area itself, and due to the challenging climb up the stairway, many visiting swimmers may either be unable or unwilling to access the restroom in the parking area at the top of the cliff.

Routine testing by the BECQ shows that enterococci levels at the Grotto frequently exceed the water quality criteria for the CNMI. The random but high densities of visiting swimmers at this site most probably have contributed to the pattern of FIBs observed there. While HF183 was not the only fecal marker detected in water samples from the Grotto, it was the dominant marker detected, although seabirds were also significant contributors to fecal contamination in the region. It is likely that bacterial shedding from the swimmers represents a primary source of the human-source fecal bacteria MST markers detected. Previous studies by others and ourselves have shown that bathers can shed large amounts of fecal and skin-associated bacteria, including *Enterococcus*, fecal *Bacteroides*, and *Staphylococcus aureus* among others (Gerba, 2000; Elmir et al., 2007, 2009; Plano et al., 2011). While there are undoubtedly some incidents of direct defecation into Grotto waters by certain individuals unwilling to hike back up the cliff to the only restroom facility, this human fecal marker signal is more likely attributable to unintentional routine bacterial shedding by general bathers. Previous studies have shown that bathers can potentially release large amounts of enterococci into the surrounding water just by normal bathing activities or even just soaking quietly, and that the degree of this bacterial shedding is highly variable between individuals and over time, and that some individuals may even be “super-shedders.” Young children and toddlers especially can also potentially shed inordinately high levels of FIB when bathing or playing in water. Both pool studies and a beach epidemiological study where participants were directed to sample their own personal water space while bathing have demonstrated bacterial shedding phenomenon by bathers (Elmir et al., 2007, 2009; Shibata et al., 2010; Sinigalliano et al., 2010). The combination of large crowds of bathers in a relatively small water body with poor circulation, or limited flushing at a particular time may lead to elevated FIB levels with the crowd of people themselves as a point source. The high-density crowds at the Grotto have also been observed to potentially represent other physical health and safety risks, particularly during the descent and ascent of large numbers of people along the steep stairway leading from the parking area to the waters of the Grotto. More robust crowd control to limit bather density at any given time, along with allowing for periods of low or closed activity to permit the natural tidal flushing of local waters, are all measures that might serve to improve both general safety and water quality at this popular and unique Saipan attraction. Additional restroom facilities that are protected from discharge or leakage to coastal waters might also be considered.

Since the time of this qPCR MST study reported here, a new on-going study of the Grotto area has been initiated by a NOAA Coral Fellow working with the BECQ. This study is looking at enterococci exceedances compared to the daily bather numbers at the Grotto, with the idea of examining thresholds of user capacity that may trigger enterococci exceedances. This study has been conducted through the impacts of both Super Typhoon Tutu and the COVID-19 pandemic. Preliminary data through July 2020 shows that the percentage of Enterococci sample exceedances at the Grotto have been reduced by half from the 2019 levels and have decreased more than threefold from the 2018 levels. It is believed that this reduction may be attributed to extensive flushing of the Grotto area by Super Typhoon Yutu and to the substantially reduced tourism following Super Typhoon Yutu and the COVID-19 pandemic. The Grotto has been closed to the public since March 2020. This is still preliminary data, and the final results of the Grotto bather usage study will be published elsewhere (unpublished data, personal communication LM, BECQ). However, these later observations from the Grotto about the reductions in enterococci levels during the COVID-19 pandemic and beach closures are consistent with the human-marker MST data presented here, and further support the idea that bather shedding is a primary input of FIBs in the Grotto area.

Bird fecal input appeared to be a major contributor to other observed enterococci hotspots, with areas such as Bird Island and American Memorial Park Drainage routinely having regulatory enterococci exceedances and high levels of Gull2 seabird marker. No other significant sources of fecal marker were found in the water samples from Bird Island; it is therefore logical to assume that bird populations are the primary cause for the chronic enterococci water quality failures of this area. Based on our Gull2 qPCR results, birds also appeared to be major fecal contributors to other areas of chronic enterococci exceedance, such as Jeffrey’s Beach and Old Man by the Sea. Thus, birds would appear to be significant contributors of FIB at areas such as the American Memorial Park Drainage, the Lao Lao Bay area, the Grotto, Jeffrey’s Beach, Old Man by the Sea and, of course, Bird Island. Such bird fecal contamination is probably not manageable in a practical sense. However, the potential of bird fecal contributions and their relatively lower risk, should be considered when assessing observed exceedances of general fecal indicators such as enterococci or *E. coli*, especially as bird fecal contributions may serve to confound general water quality assessments. If significant bird contribution is determined to be a primary contributing factor in recreational water sites of chronic exceedance without any other high-risk fecal input sources, alternative site-specific water quality criteria might be considered. In this case, a combination of methods, including MST and Quantitative Microbial Risk Assessment, could be deployed to help confirm if any such alternative criteria promulgated by the BECQ would likely be equally protective of both public and environmental health.

In examining coral reef exposure, this study found that LBSP-derived microbial contaminants were being transported from the Saipan Lagoon coastline to the reef tract. Therefore, the reefs offshore from the Saipan Lagoon coast may also potentially be exposed to pathogens and other contaminants associated with this LBSP pollutant transport (likely including microbial, nutrient, and chemical contaminants as well). While reef waters were tested during this study, coral tissues were not. Consequently, it could not be determined whether microbial contaminants were being absorbed into or influencing the coral holobionts at these reefs. However, impacts of LBSP microbial contaminants on coral microbiota have been documented at other coral reefs, including reefs in southeast Florida (Staley et al., 2017). A follow-up study to test coral tissue along the Saipan reef tract for the presence of fecal indicator markers and/or pathogens would be useful to further define reef exposure. From the pattern of results of this MST source tracking study, it would appear the most likely source of the exposure and impact to these reef sites is the western central Saipan Lagoon shoreline area, particularly the region from roughly sites S17 through S22 (i.e., roughly from the area of the Garapan Fishing Dock to Susupe).

Based on this MST study, the following recommendations have been made to the resource managers of the CNMI-BECQ:

(1) Conduct further investigations of sanitary infrastructure and potential leakages (including further investigation of potential groundwater contamination), especially along the west central Saipan Lagoon shoreline, and aggressively pursue the detection and repair of sanitary infrastructure in the region.
(2) Conduct further investigations of the potential contamination of stormwater runoff, especially in the regions of Tanapag, Garapan district (including American Memorial Park), Susupe, and the west central Saipan Lagoon shoreline.
(3) Implement more robust control measures for dealing with animal waste, particularly dog fecal contamination, especially along the western coast of Saipan Lagoon. A large number of dog fecal marker elevations suggest that dog fecal influence is widespread. Among other things, consider mandating dog fecal cleanup policies and provide the necessary resources to make dog fecal cleanup in public spaces easy and cost effective.
(4) Implement more robust bather crowd control measures at high density bathing beaches and recreational water areas, especially at the Grotto, along with more accurate bather density monitoring of crowds at the Grotto. Managing crowd size at the Grotto will allow more time for natural tidal flushing, which might be advantageous to maintaining better water quality at the site. It would also likely provide a benefit in increased physical safety and reduced accidents.
(5) Integrate periodic molecular MST assessments into the more routine water quality monitoring efforts of the BECQ to permit a better understanding of the seasonality, infrastructure, environmental, and social factors that influence source contributions of contaminants to local waters, and to help trouble-shoot any sites of chronic exceedances. BECQ is now also planning to expand MST assessments to other less populated islands of CNMI, including Tinian and Rota, where it is suspected that the predominant FIB sources may be dogs and birds, rather than humans as in Saipan (personal communication, LM, BECQ).
(6) Further test the CowM2 and Pig2Bact qPCR assays to determine whether local or regional factors (e.g., livestock diet, etc.) affect the detection sensitivity of these two livestock assays. If this turns out to be the case, modified or alternate MST protocols for livestock could be developed or adopted to boost the detection sensitivity for local Saipan livestock fecal contamination. Also, supplement testing with the use of a more general ruminant fecal marker such as the Rum2Bact assay.

The results from this study demonstrate the value of integrating molecular MST technology into the assessments of water quality in the CNMI, along with providing the first baseline assessment of host-specific fecal water quality indicators for the CNMI region.

## Conclusion

In conclusion, we identified a need for molecular MST capability within CNMI regulatory agencies. We initiated an MST technology transition effort between NOAA-AOML and the CNMI-BECQ and used this new MST capacity at the BECQ to conduct the first baseline assessment of host-specific fecal water quality microbial indicators in the coastal waters and protected coral reefs of Saipan. We found that the west central shoreline region of Saipan Lagoon was the greatest contributor of human-specific fecal bacteria contamination to the lagoon and reef tract during the period of study. Our study also identified another hotspot of human fecal marker contamination at the popular recreational swimming and scuba diving site of the Grotto. We have suggested that the primary input of fecal bacteria for the Saipan Lagoon is most likely compromised sanitary infrastructure along the western Saipan Lagoon shoreline from Garapan Fishing Dock to Susupe, while at the Grotto the most likely cause is the high density of human bathers.

Birds also appeared to be significant contributors of FIB to areas such as the American Memorial Park Drainage, the Lao Lao Bay area, the Grotto, Jeffrey’s Beach, Old Man by the Sea, and, of course, Bird Island. Such bird fecal input is probably not manageable in a practical sense, but it should be recognized that bird fecal contributions may serve to confound general water quality risk assessments such as the use of general culture-based enterococci tests. We also detected much less fecal marker associated with pigs and cows than expected, but we suspect the particular qPCR assays used (CowM2 and Pig2Bact) might have a lower sensitivity to the specific populations of Saipan livestock. We recommend that further investigation and optimization of MST methods for these specific populations of Saipan livestock may be warranted, as well as use of more general ruminant fecal qPCR assays. This study also found that moderate levels of dog fecal contamination were widespread on the island and have suggested a more robust control of animal fecal waste management. Overall, we believe the inclusion of molecular MST capability as demonstrated here can be a useful addition to the water quality toolbox of resource managers in the CNMI to enhance the ability of the BECQ to better protect public health and that of the environment.

## Data Availability Statement

The original contributions presented in the study are included in the article/Supplementary Material, further inquiries can be directed to the corresponding author/s.

## Author Contributions

CS conceived and designed the experiments, performed the experiments, contributed reagents, materials, and analysis tools, analyzed the data, prepared the figures and/or tables, authored or reviewed drafts of manuscript, approved the final draft, and serves as Principal Investigator and as corresponding author. KK conceived and designed the stable nitrogen isotope experiments, participated in sampling, analyzed the data, prepared the figures, authored or reviewed drafts of manuscript, and approved the final draft. MG conceived and designed the experiments, performed the experiments, contributed reagents, materials, and analysis tools, analyzed the data, prepared the figures and/or tables, authored or reviewed drafts of manuscript, and approved the final draft. KY conceived and designed the experiments, analyzed the data, prepared the figures and/or tables, authored or reviewed drafts of manuscript, and approved the final draft. KKn conceived and designed the SGD experiments, participated in sampling, analyzed the data, authored or reviewed drafts of manuscript, and approved the final draft. DP performed the experiments, contributed reagents, materials, and analysis tools, reviewed drafts of manuscript, and approved the final draft. CB performed the experiments, contributed reagents, materials, and analysis tools, authored or reviewed drafts of manuscript, and approved the final draft. AB performed the experiments, analyzed the data, reviewed drafts of manuscript, and approved the final draft. HL performed the experiments, analyzed the data, reviewed drafts of manuscript, and approved the final draft. LM provided oversight for BECQ activities for the project and the manuscript, contributed materials and analysis tools, reviewed drafts of manuscript, and approved the final draft.

## Disclaimer

Neither the United States Government nor any of its employees, contractors, or their employees make any warranty, expressed or implied, or assumes any legal liability or responsibility for any third party’s use of apparatus, product, or process discussed in this document, or represents that its use by such party would not infringe on privately owned rights. Mention of any commercial entities, trade names, or commercial products in this document does not constitute any type of endorsement or recommendation for use.

## Conflict of Interest

The authors declare that the research was conducted in the absence of any commercial or financial relationships that could be construed as a potential conflict of interest.
