# Supplementary material for: Microbial Source Tracking of Fecal Indicating Bacteria in Coral Reef Waters, Recreational Waters, and Groundwater of Saipan by Real-Time Quantitative PCR
Source: Front Microbiol. 2021 Jan 18;11:596650. doi: 10.3389/fmicb.2020.596650 (PMC7848096; doi:10.3389/fmicb.2020.596650)
Supplement: Supplementary file 1 [file Data_Sheet_1.pdf]

Supplementary Material

Sinigalliano et al., 2020, Frontiers in Microbiology
Microbial Source Tracking of Fecal Indicating Bacteria in Coral Reef Waters, Recreational Waters, and Groundwater of Saipan by Real-Time Quantitative PCR

Supplemental information on Methods and Supplemental Tables S1 to S4

Supplemental Information on Materials and Methods:

Sample filtration and environmental DNA extraction:

At the BECQ laboratory, aliquots of sample were filtered through sterile 0.45 µm pore-size, 47 mm diameter cellulose ester filters (Pall GN6, Pall Corporation) using disposable sterile filter funnels (Pall Microfunnel, Pall Corporation). Water samples were filtered up to 1 L volume or until filter clogging. In some cases a smaller volume was filtered for samples where the water was too turbid to allow for the filtration of 1 L, or in the case of ground water where a smaller volume was collected. The final sample volumes ranged from <100 mL to 1 L. In all cases, the actual volume filtered was recorded (as shown in Supplemental Table S5) and used in subsequent calculations for MST target copy number per given sample volume. For the BECQ training demonstration samples used in September 2017 and for groundwater samples used in August 2018, the water samples were filtered through sterile 0.4 µm pore-size, 47 mm diameter polycarbonate filters that came pre-loaded as part of the Pall disposable MicroFunnels (Pall Corporation), since those filter/funnel assemblies were already available at the BECQ laboratory. These pre-loaded polycarbonate filters were primarily used for the small-scale preliminary sampling that was conducted for training demonstrations with the BECQ staff. The regular MST baseline sampling conducted in 2018 utilized the cellulose ester filters, consistent with previous NOAA MST studies of marine waters (Sinigalliano et al, 2010; Campbell et al, 2015; Symonds et al; 2016; Staley et al, 2017; Sinigalliano et al, 2019). Sample filters were rolled using flame-sterilized forceps and aseptically transferred to Lysing Matrix E bead beat tubes (from the FastDNA Spin Kit for Soil, MP Biomedicals). Due to a lack of available -80°C freezer facilities at Saipan, the filters in these tubes were then preserved by adding approximately 2 mL of DNAgard Tissue preservative solution (Biomatrica) and stored frozen in a standard -20°C freezer until later DNA extraction and purification.

For the 12 MST workshop training demonstration samples collected in September 2017, extraction and purification of total genomic DNA was performed by the GeneDisc Ultra-Purifier Extraction System protocol (Pall Corporation) as per the manufacturer's instructions for the GeneDisc Recreation Water *E. coli* and *Enterococcus* spp. assay kit (Pall Corporation). This was conducted using the Pall Extractor System that was already in the BECQ lab. In brief, the sample filter was aseptically transferred to a lysis tube from the extraction kit using flame-sterilized forceps, then sonicated in the Pall kit lysis buffer and heated at 110°C for 20 min. The lysate was filtered under vacuum onto a silica DNA-binding column from the kit in the Pall Ultra-Purifier instrument to bind the DNA and washed twice with the kit washing buffers #1 and #2 under vacuum. The column was dried, and the bound purified DNA was eluted from the silica binding column with a total of 200 µL of pre-heated elution buffer from the kit. The eluted

DNA was collected under vacuum into the final DNA recovery tubes from the kit, and the purified genomic environmental DNA extract was stored frozen at -20°C until analysis.

For all of the other Saipan MST study water samples collected between March and August of 2018 and stored on 0.45 µm cellulose ester filters, the extraction and purification of total genomic DNA was performed by the FastDNA Spin Kit for Soil protocol (MP Biomedicals, Thermo-Fisher) and using a SuperFastPrep-2 bead-beating homogenizer instrument according to the manufacturer's instructions with minor modifications as follows. This filtration and extraction procedure was utilized instead of the previous Pall Extractor system for the regular MST baseline study in 2018 to again be consistent with previous MST studies conducted by NOAA in the marine environment. Filters were stored until processing in Lysing Matrix E bead tubes from the kit (MP Biomedicals) filled with DNAgard Tissue preservative solution (Biomatrica) as described above. For later extraction, in the case of frozen filters, the samples preserved in the bead-beat tubes were first completely thawed. Tubes were centrifuged down for 5 minutes as 12,000 x g, and the majority of the DNAgard preservative solution was gently pipetted off and discarded without disturbing the filter, beads, or cell pellet. The rest of the extraction processing was as per kit instructions. In general, 978 µL of the kit sodium phosphate buffer and 122 µL of the kit MT lysis buffer were added to the bead-beat tubes with the filter, and the cell samples on the filter were then lysed and homogenized by vigorous bead beating using the hand-held beat-beating Super FastPrep-2 homogenizer instrument (MP Biomedicals) as per the manufacturer's instructions at a speed setting of "20" for a duration of 5 seconds. According to the manufacturer, this gives an equivalent bead-beating homogenization as compared to an impact speed of 6 m/s for 60 seconds in the FastPrep-24 benchtop instrument. The homogenized lysates were centrifuged at 12,000 x g for 15 minutes to pellet beads and cell debris, the lysate was then transferred to a new tube for protein precipitation with the kit's PPS buffer, and centrifuged again at 12,000 x g for another 5 minutes to pellet precipitated protein. The exogenous DNA from the cleared lysate supernatant was bound to the kit's Binding Matrix column, washed twice with the kit's wash buffer, and eluted with the kit's elution buffer (warmed to 55°C for 5 min.). Binding columns were eluted with a total of 80 µL of elution buffer and collected by centrifugation for 2 min at 12,000 x g into the kit recovery tubes. The eluted DNA was then stabilized for room temperature storage by the addition of 20 µL of DNA-Stable-Plus preservative solution (Biomatrica), giving a final extracted elution volume of 100 µL. These elutions were subdivided into replicate aliquots and stored frozen in a standard -20°C freezer for later analysis. Replicate aliquots were also transported at room temperature back to the NOAA-AOML lab in Miami, Florida for later performance validation comparisons between the BECQ GeneDisc instrument (Pall) and the AOML StepOnePlus instrument (Applied Biosystems) as part of the broader BECQ technology transfer performance assessment. Total DNA extraction yields for all samples were quantitated on the Qubit 3.0 fluorometer system (Invitrogen) with the Quant-iT HS dsDNA assay kit (Invitrogen) as per manufacturer's instructions.

Modification of MST qPCR assays for GeneDisc platform:

The established molecular MST protocols described in Griffith et al. (2013) have typically been deployed using research-grade quantitative real-time PCR thermocyclers that are highly customizable. A variety of these platforms have been tested and validated in a number of multi-lab trials (Boehm et al., 2013). However, some modifications were needed to adapt these

protocols to the format of the Pall GeneDisc qPCR Rapid Microbiology System thermocycler that was available in the BECQ laboratory. This Pall GeneDisc system was designed to be simple and user friendly, but by its nature is not typically customizable. Rather than being a research-based thermocycler, it is a specialized but easy-to-use commercial applications thermocycler primarily intended for the food and beverage quality control industry, but is now being more widely introduced for water quality applications. As such, the GeneDisc thermocycler is a highly automated system designed to run pre-prepared GeneDisc plate kits with all reagents, primers, probes, and other assay components already pre-loaded by the company and preserved in the plate wells. End-users of the GeneDisc system simply add a DNA sample to the plate and scan a bar code to initiate analysis, which automatically conducts pre-programmed assay cycling parameters, and automated analysis and interpretation of the results to make a simple end-user report as output. End-users of this system typically cannot modify or adjust the cycling program, cannot adjust analyses or standard curves, nor can they change or add their own reagents; they can only run pre-made assay kits provided by the company and let the instrument do automated analyses for that specific kit. There are commercial GeneDisc kits available for water quality measurements of general enterococci and *E. coli* (which the BECQ lab had already been using). However, the Pall Corporation had no available GeneDisc kits for any host-specific MST markers at the time of this study while we were conducting our associated NOAA technology transition project of MST methods to the CNMI BECQ.

In support of our MST technology transition from NOAA to BECQ, the GeneDisc Division of Pall Corporation graciously custom modified the software of the particular GeneDisc instrument at the BECQ lab and specifically provided custom blank "open" MST GeneDisc plates without pre-loaded reagents. This allowed BECQ personnel to run their own thermocycling parameters and conditions, as well as load their own MST reaction cocktails as per the standard MST protocols in the appendices of the California Microbial Source Identification Manual (Griffith et al., 2013). The specific MST assays used for the Saipan microbial source tracking study included: (1) human-source *Bacteroides* assay HF183 (EPA Taqman MGB version); (2) dog-source Bacteroidales assay DogBact; (3) cow-source Bacteroidales assay CowM2; (4) pig-source Bacteroidales assay Pig2Bact; and (5) the Gull2 assay specific for *Catellicoccus marimammalium* fecal bacteria found in the gut of most seagulls, as well as potentially in the gut of other birds (especially seabirds) that may co-habit, scavenge, or nest with seagulls. Depending on the specific geographic location and co-nesting behavior, this may also include species of terns, pelicans, geese, and very often, pigeons (Sinigalliano et al., 2013). The sequences of the oligonucleotides for primers and probes, as well as the synthetic dsDNA standard control fragments, are listed in Supplemental Table S3.

Quantitative PCR reactions for these MST assays were configured as per the California Microbial Source Identification Manual (Griffith et al., 2013) with the following modifications: (1) Total reaction volumes were 12 µL per plate well, set up as sectors for three plates, where 30 µL of working reaction cocktail were mixed with 6 µL of target sample DNA and pipetted into the respective sample sector of a blank GeneDisc plate type 01MT, which then filled three replicate plate wells under vacuum with 12 µL of the sample/reaction cocktail mixture. (2) All assays were modified and optimized to work in the final PCR reaction at a final forward and reverse primer concentration of 1 µM. (3) Final probe concentrations were 80 nM. (4) Proprietary Pall Corporation 1X GeneDisc Mastermix (Pall Cat# SR008) was used in all qPCR reactions. The resulting qPCR reaction cocktails were sealed in the GeneDisc wells with sterile mineral oil as per the manufacturer's instructions. The thermocycler was then run with the

proprietary Pall Genefile plate file 01MT_0B that is specific for the custom run of the blank MST GeneDiscs type 01MT with cycling conditions of 15 minutes at 95°C to activate the hotstart polymerase enzyme of the mastermix, followed by 40 repetitive cycles of the 95°C denaturation step for 15 sec, followed by the 60°C annealing and extension step for 1 min, with a fluorescence reading of FAM and ROX dye in each well at the end of each extension step. The baseline and fluorescence cycle threshold (CT) were set to automatic by the instrument. Triplicate standard curves used to convert Cq values (the fraction of cycles for each reaction where the amount of fluorescence reaches the set cycle threshold in the log phase of the amplification) into the calculated Copy Number of sequence target per well were constructed from dilutions of a known copy number of synthetic double-stranded target DNA standards (gBlocks, synthesized by Integrated DNA Technologies), ranging from 10^5 to 10^1 gene copies per reaction well for the standards.

The instrument recorded the Cq cycle values from the raw fluorescence CT values. Since the GeneDisc instrument software was not designed to automatically generate standard curves and perform quantity calculations for these particular custom blank MST assay plates with the on-board instrument software (as it normally would with the regular GeneDisc commercial kits), the fluorescent read data and Cq/CT data were exported as .csv files from the instrument and imported into Microsoft Excel in spreadsheet format. Linear regression standard curves of the Cq values versus the Log10 value of the standard DNA concentrations were plotted with the SigmaPlot ver 14 statistical graphing and analysis software package (Systat Software, Inc.). The final mean quantities from the three replicate wells for each environmental sample or control sample were then calculated in target sequence copies per reaction based upon the sample Cq value and the slope and intercept of the linear regression of the target DNA positive control concentration standards. The quantities in copy number for the environmental samples were then determined by comparing their Cq values to this positive control standard curve to determine copies per reaction, then adjusted for the sample filtration, dilution, and elution volumes to give the final copies per 100 mL of water sample.

Quantitative PCR quality assurance and controls:

MST qPCR assay quality assurance procedures and controls are as described in EPA Method 1696 for the characterization of human fecal pollution in water by HF183/BacR287 Taqman qPCR (US EPA, 2019). These QA/QC metrics were used as guidance for the QA/QC assessment of all the assays. The standard curve quality control metrics for these qPCR assays as run on the BECQ lab's Pall GeneDisc instrument Saipan are shown in Supplemental Table S4. The lower limit of quantitation (LLOQ) was determined from the standard curves of each batch run, where the LLOQ of the reactions was determined from the 95% prediction upper limit of the 1 $\log_{10}$ copy DNA standard dilution for the triplicate standard curves of each batch run. The LLOQ values for each assay are also displayed with the standard curve QA/QC metrics shown in Supplemental Table S4.

Samples with a Cq value greater than the reaction LLOQ were categorized as "DNQ" or "detected but not quantifiable." To estimate an approximate environmental LLOQ from the actual assay reaction LLOQs, and based on the standard curve statistics for all batch runs, the overall reaction sensitivity of the LLOQ for this batch of standard curves was taken to be equivalent to 10 target sequence copies per GeneDisc reaction well for each of the MST assays used in this study. The average reaction LLOQ for all combined assays was 10.41 copies/rxn

with a range of 8.91 to 12.99 copies/rxn (Supplemental Table S4). Therefore, the equivalent environmental water sample detection sensitivity of this batch LLOQ would be 50 target sequence copies per 100 mL of water sample (given that 1 L water samples were filtered, extracted, and eluted into 100 µL of pure DNA and then 2 µL of DNA elution used per reaction well). This means that water sample MST values below 50 copies per 100 mL of the environmental LLOQ threshold for a particular MST target should be considered to be in the category of "detected but not quantifiable", or "DNQ", and therefore considered as insignificant for target abundance in the environmental sample. Samples were judged as "ND" or "not detected" when fluorescence intensity values for the reaction did not reach the CT within 40 cycles, and therefore a quantitation threshold Cq value could not be determined. The qPCR runs had an efficiency of between 90% and 110% as required by EPA QA/QC, except for the seabird Gull2 assay which only had an amplification efficiency of 81% for these batch of standard curves. While this is not desirable, and under normal circumstances would suggest a replacement of the primer/probe stocks for that assay and repeat of analysis, this was not a possibility in Saipan for this particular study during this particular time frame. We acknowledge that this may cause an underestimation of the actual level of bird signal in these particular samples, but this still provides some useful data in context of the other marker assay data, and does not suggest a change in the overall conclusions of the study. All run standard curves for all assays had an $R^2 \geq 0.98$, so all other QA/QC metrics for the standard curves were satisfied (with the already acknowledged exception of the amplification efficiency for the bird marker).

Negative qPCR controls consisted of both "no template controls" (NTC) where no target DNA was added to the reaction wells and method blank (MB) controls where sterile water negative control samples were filtered, extracted, and analyzed in the same manner as the environmental water samples. For the most part, all negative controls were ND, not detected. However, some negative controls for the DogBact marker showed very low levels of detection, but such detections of the DogBact NTCs or method blanks had Cq cycle values substantially higher than the determined the LLOQ Cq cycle (i.e., all detected negative controls for DogBact had MST quantitation values for target copies per reaction that were much lower than the LLOQ quantitation value and were far down in the determined DNQ range, even though there was still a technical positive detect of the negative control by the instrument). The environmental assay results based on these batch runs for the DogBact assay were judged to still be valid following the QA/QC guidelines of EPA Method 1696, which states "…although no [negative control] reaction should yield a Cq value, Cq values greater than the LLOQ are acceptable for quantification applications only. However, the laboratory should report this practice" (US EPA, 2019, pp.15), as was done here for the DogBact assay samples.

For sample processing controls (SPC), the variability in sample processing efficiency or inhibition was measured for each environmental sample and method blank sample by using a spike preparation consisting of a fixed concentration of salmon DNA by adding purified salmon testes DNA, (Sigma cat# D7656) directly into the extraction lysis buffer used for filter extractions at a final concentration of 0.2 ug/mL, with all sample extractions (environmental samples and method blanks) getting the same amount of the SPC salmon DNA. Each sample was then analyzed in a separate set of assay reactions for the Sketa22 qPCR assay targeting the ITS region 2 of chum salmon (Griffith et al., 2013; US EPA, 2019). The Sketa22 Cq values for the environmental samples were then compared to the Cq values of the method blanks (that had no MST target DNA but did have the same amount of salmon SPC DNA). The SPC acceptance threshold for the environmental samples was then determined by the Cq plus three standard

deviations for the mean of the Sketa22 assay values for the three method blanks of each batch as per EPA Method 1696 (US EPA, 2019).

Samples that failed SPC criteria were diluted 1:10 and re-run with both the MST target assay and the Sketa22 assay, while comparing the Sketa22 Cq value to the mean of method blank reactions from the batch that had been similarly diluted. Samples still outside of the acceptance threshold were then deemed to be either too inhibited or outside of the accepted extraction efficiency range and were subsequently removed from the data set. Only two environmental samples required elimination in this manner.

Statistical boxplot analyses of the MST result patterns for host-specific fecal bacterial markers were generated with the SigmaPlot software package, version 14 (Systat Software, Inc.). Final MST positive control concentration standard curves and their associated linear regression statistics were also plotted using the SigmaPlot v.14 software package.

Supplemental Table S1: GPS coordinates for the Saipan MST study sample sites collected by BECQ personnel from their regular Water Quality Surveillance Program sample sites. Site labels correspond with the regular BECQ water quality surveillance site IDs.

Sample Site ID	Latitude decimal degrees	Longitude decimal degrees	BECQ Water Quality Program Site Description
NEB01	15.25872359	145.82319891	Grotto
NEB02	15.25956572	145.81402146	Bird Island
NEB03	15.22544400	145.79102700	Jeffrey's Beach
NEB04	15.20973484	145.77922334	Old Man by the Sea
NEB07	15.13320000	145.47240000	Hidden Beach
WB07	15.24269986	145.75359491	Tanapag Meeting Hall
WB08	15.23218678	145.74155397	Central Repair Shop
WB10	15.22625876	145.73769149	DPW Channel Bridge
WB12.1	15.21811662	145.72048989	American Memorial Park Drain
WB16	15.21447578	145.71547442	Dai Ichi Hotel
WB17	15.21323831	145.71556180	Drainage #1
WB19	15.20955485	145.71544512	Hafa-Adai Hotel
WB20	15.20882265	145.71538005	Drainage #2
WB21	15.20218380	145.71586838	Garapan Fishing Dock
WB22	15.19647489	145.71667415	Garapan Beach
WB23	15.19946847	145.71630856	Drainage #3
WB30	15.15161651	145.69991513	Sugar Dock
WB31	15.14827499	145.70005013	CK Dist #2 Drain
EB02	15.16256974	145.76436653	North Lao Lao Beach
SEB03	15.16084846	145.75497900	South Lao Lao Beach
SEB05	15.10665252	145.71725701	Ladder Beach

Supplemental Table S2: GPS coordinates for the Saipan MST study sample sites collected by American University personnel during their nitrogen isotope source tracking study. Site labels correspond with American University study site IDs.

Lagoon Region and Sample Site ID	Latitude decimal degrees	Longitude decimal degrees
Tanapag - S01	15.272307	145.792983
Tanapag - S02	15.268146	145.787746
Tanapag - S03	15.262718	145.78402
Tanapag - S04	15.256746	145.781119
Tanapag - S05	15.252371	145.776126
Tanapag - S06	15.250323	145.769663
Tanapag - S07	15.245921	145.764582
Tanapag - S08	15.243969	145.758113
Tanapag - S09	15.240486	145.75242
Tanapag - S10	15.235147	145.746218
Tanapag - S11	15.231979	145.741222
Tanapag - S12	15.22641	145.738477
Tanapag - S13	15.221355	145.726457
Tanapag - S14	15.219648	145.719871
Garapan - S15	15.214674	145.715417
Garapan - S16	15.208152	145.715918
Garapan - S17	15.201604	145.716251
Garapan - S18	15.195086	145.71668
Garapan - S19	15.18867	145.715166
Garapan - S20	15.182211	145.713697
Garapan - S21	15.175859	145.711938
Garapan - S22	15.169579	145.70999
Garapan - S23	15.163536	145.707222
Garapan - S24	15.158366	145.701672
Garapan - S25	15.152443	145.70056
Chalan Kanoa - S26	15.145947	145.69951
Chalan Kanoa - S27	15.139724	145.697355
Chalan Kanoa - S28	15.133885	145.694066
Chalan Kanoa - S29	15.126143	145.693372
Reef - R02	15.254677	145.770817
Reef - R03	15.252171	145.740146
Reef - R04	15.251834	145.723624
Reef - R05	15.238982	145.703865
Reef - R06	15.217466	145.706273
Reef - R07	15.200849	145.708484
Reef - R08	15.183176	145.702492
Reef - R09	15.170759	145.699209
Reef - R10	15.150247	145.696933
Lao Lao Bay - L01	15.162799	145.765156
Lao Lao Bay - L02	15.163437	145.760804
Lao Lao Bay - L03	15.161266	145.754351
Lao Lao Bay - L04	15.159449	145.75032

Supplemental Table S3: Primers, Probes, and gBlock positive control sequences used for qPCR assays in this study.

**Host /<br>Assay Name**	**Oligonucleotide Primer/Probe sequences<br>& gBlock Positive Control sequences*<br>(sequence 5'→ 3')**
**HUMAN:**<br>HF183 Taqman<br><br>(EPA version<br>HF183/BacR287)	**Target organism: Genus** *Bacteroides* - 16S rRNA gene cluster . **REF:** Griffith et al (2013); US-EPA Method 1696 (2019).<br>Forward Primer: HF183. ATCATGAGTTCACATGTCCG<br>Reverse Primer: BacR287. CTTCCTCTCAGAACCCCTATCC<br>Probe: BacP234MGB. [6FAM]-CTAATGGAACGCATCCC-[NFQ-MGB]<br>HF183 gBlock positive control:<br>ATCATGAGTTCACATGTCCGCATGATTAAAGGTATTTTCCGGTAGACGATGGGGATGCGTTCCATTAGCTCGAGATAGTAG<br>GCGGGGTAACGGCCCACCTAGTCAACGATGGATAGGGGTTCTGAGAGGAAGG
**DOG:**<br>DogBact	**Target organism: Order** Bacteroidales - 16S rRNA gene. **REF:** Griffith et al (2013); Schriewer et al (2013).<br>Forward Primer: DF475F. CGCTTGTATGTACCGGTACG<br>Reverse Primer: Bac708R. CAATCGGAGTTCTTCGTG<br>Probe: DogBactP. [6FAM]-ATTCGTGGTGTAGCGGTGAAATGCTTAG-[BHQ1]<br>DogBact gBlock positive control:<br>CTTTTGTCCGGGAATAAAACCGCCTACGTGTAGGCGCTTGTATGTACCGGTACGAATAAGCATCGGCTAACTCCGTGCCAG<br>CAGCCGCGGTAATACGGAGGATGCGAGCGTTATCCGGATTTATTGGGTTTAAAGGGAGCGCAGACGGGTTTTTAAGTCAG<br>CTGTGAAAGTTTGGGGCTCAACCTTAAAATTGCAGTTGATACTGGAGACCTTGAGTGCAGTTGAGGCAGGCGGAATTCGT<br>GGTGTAGCGGTGAAATGCTTAGATATCACGAAGAACTCCGATTGCGAAGGCAGCTTGCTAAAGTGTAACTGACGTTCATG<br>CTCGAAAGTGTGGGTATCAAACAGGATTAGATACCCTGG
**PIG:**<br>Pig2Bac	**Target organism: Order** Bacteroidales - 16S rRNA gene. **REF:** Griffith et al (2013); Mieszkin et al (2009).<br>Forward Primer: Pig-2-Bac41F. GCATGAATTTAGCTTGCTAAATTTGAT<br>Reverse Primer: Pig-2-Bac163Rm. ACCTCATACGGTATTAATCCGC<br>Probe: Pig-2-Bac113P [6FAM]-TCCACGGGATAGCC-[NFQ-MGB]<br>Pig2Bac gBlock positive control:<br>TCCAAACTCCTACGGGAGCGTCAGGTTTGTTTCGGTATTGAGTATCGAAAATCTCACGGATTAACTCTTGTGTACGCTCTC<br>GAGGACCAGCTAATGCATATAAATAAGTTACGTGATGACGGCCAAATACTCCTGATCGTACTCGAGATAGGCACCTATGT<br>CCTTTACCTCATCAACTACAGACAAAATTATCTCAAGGAACGCAACAAGCCCTCTAATGGAAAATGGATGGTATCTTTGG<br>AGCCTTTGAAAGCACTCGAGCCTTATGCATTGAGCATCGAGGCCGGAAAGCAGGAACTTATATATAATAAGGTATTAGCA<br>GGCGAAGTATGGATGGCTTGCTAAATTTGATGGCGACCGGCGCACGGGATCCTAACGCGTATCCAACCTTCCCTTATCCAC<br>GGGATAGCCCGTCGAAAGGCGGATTAATACCGTATGAGGTGCGGCCGCGGATCGACGAGAGCAGCGCGACTGGATCAGT<br>TCTGGACGAGCGAGCTGTCGTCCGACCCGTGATCTTACGGCATTATACGTATGATCGGTCCACGATCAGCTAGATTATCTA<br>GTCAGCTTGATGTCATAGCTGTTTCCTGAGGCTCAATACTGACCATTTAAATCATACCTGACCTCCATAGCAGAAAGTCAA<br>AAGCCTCCGA

**COW:**<br>CowM2	**Target organism: Order** Bacteroidales - functional gene that putatively encodes for membrane-associated and secreted proteins involved in energy metabolism and electron transport **REF:** Griffith et al (2013); Shanks et al (2008).<br>Forward Primer: CowM2F. CGGCCAAATACTCCTGATCGT<br>Reverse Primer: CowM2R. GCTTGTTGCGTTCCTTGAGATAAT<br>Probe: CowM2P. [6FAM]-AGGCACCTATGTCCTTTACCTCATCAACTACAGACA –[BHQ1]<br>CowM2 gBlock positive control:<br>TAAATAAGTTACGTGATGACGGCCAAATACTCCTGATCGTACTCGAGATAGGCACCTATGTCCTTTACCTCATCAACTACA<br>GACAAAATTATCTCAAGGAACGCAACAAGCCCTCTAATGGAAAATGGAT
**SEABIRD:**<br>Gull2	**Target organism:** *Catellicoccus marimammalium* - 16S rRNA gene. **REF:** Griffith et al (2013); Sinigalliano et al (2013).<br>Forward Primer: Gull2F TGCATCGACCTAAAGTTTTGAG<br>Reverse Primer: Gull2R GTCAAAGAGCGAGCAGTTACTA<br>Probe: Gull2P. [6FAM]-CTGAGAGGGTGATCGGCCACATTGGGACT-[BHQ1]<br>Gull2 gBlock positive control:<br>TAATACATGCAAGTCGAACGCAAAACTTTTAACTGATGCTTGCATCGACCTAAAGTTTTGAGTGGCGGACGGGTGAGTAA<br>CACGTGGGTAACCTGCCCATCAGAGGGGGACAACACTTGGAAACAGGTGCTAATACCGCATAATACAGAGAACCGCATG<br>GTTCTTTGTTGAAAGGCGCTTCTGGTGTCGCTGATGGATGGACCCGCGGTGCATTAGCTAGACGGTGAGGTAACGGCTCAC<br>CGTGGCAATGATGCATAGCCGACCTGAGAGGGTGATCGGCCACATTGGGACTGAGACACGGCCCAAACTCCTACGGGAG<br>GCAGCAGTAGGGAATCTTCGGCAATGGACGAAAGTCTGACCGAGCAACGCCGCGTGAGTGAAGAAGGTTTTCGGATCGT<br>AAAACTCTGTTGTTAGAGAAGAACAGGAGCGATAGTAACTGCTCGCTCTTTGACGGTATCTAACCAGAAAGCCACGGCTA<br>ACTACGTGCCAGCAGCCGCGGTAATACGTAGGTGGCAAGCGTTGTCCGGATTTATTGGGCGTAAAGCGAGCGCAGGCGGT<br>C
**Sample Processing Control (Salmon):**<br>Sketa22	**Target organism:** *Oncorhynchus keta* (chum salmon) rRNA gene operon, Internal Transcribed Spacer (ITS) region 2.<br>**REF:** Griffith et al (2013); US-EPA Method 1696 (2019).<br>Forward Primer: SketaF2. GGTTTCCGCAGCTGGG<br>Reverse Primer: SketaR2. CCGAGCCGTCCTGGTC<br>Probe: SketaP2. [6FAM]-AGTCGCAGGCGGCCACCGT-[BHQ1]

* All primer, probe, and gBlock oligonucleotide sequences were synthesized by Integrated DNA Technologies (IDTDNA.com), except for the minor-groove-binding probes using NFG-MGB quenchers, which were synthesized by Applied Biosystems-Life Technologies (www.FisherSci.com)

Supplemental Table S4: Quality Control Parameters of qPCR for MST Assays performed on Pall GeneDisc at BECQ.

qPCR Assay	Regression equation*	R^2	*E**	LLOQ* (Cq)	LLOQ* (copies/rxn)	Environ. LLOQ (copies/100 mL water)	NTC* (Cq)	Method Blank (Cq)
Human HF183	Cq=39.115 - (3.393 * log10std)	0.991	0.97	35.892	8.91	44.55	nd	nd
Canine DogBact	Cq=38.569 - (3.363 * log10std)	0.998	0.98	35.437	8.54	42.70	>39.5	>39.5
Seabird Gull2	Cq=41.515 - (3.880 * log10 std)	0.997	0.81	37.264	12.46	62.30	nd	nd
Porcine Pig2Bact	Cq=36.101 - (3.437 * log10std)	0.996	0.95	32.295	12.80	64.00	nd	nd
Bovine CowM2	Cq=42.574 - (3.480 * log10std)	0.997	0.94	38.698	12.99	64.95	nd	nd

Average combined LLOQ for all assays = 11.14 copies/rxn; Standard Deviation combined LLOQ for all assays = 1.98 copies/rxn
Median combined LLOQ for all assays = 12.46 copies/rxn

* The quality control parameters described represent the average master standard curves of all of the replicate standard curves for a particular assay on the Pall GeneDisc instrument at the BECQ lab in Saipan, during the period of the study. The regression equation for the qPCR standard curves is expressed "Cq = intercept – (slope * log10std)", where "Cq" is the value in fractions of a cycle where the fluorescence intensity of the amplification crosses the cycle threshold ("Ct value"). The "log10std" is the average value of the all the replicate lowest log10 positive control concentration standards (i.e. 2 µL of 1log10 standard used per well) for all of the standard curves for a particular assay in copies/reaction where that concentration reaches the cycle threshold and generates a corresponding average Cq value. "E" is the amplification efficiency, calculated as "E = -1 + 10^(-1/slope)". The E value should typically be between 0.90 and 1.10. The "LLOQ Cq" value of the reaction in Cq is determined from the 95% prediction upper limit at the 1 log10 copy DNA standard dilution for the replicate standard curves of each batch. The "LLOQ copies/rxn" are the calculated average quantity values from the standard curve regression equation for the corresponding LLOQ Cq value. "NTC" are "No Template controls" containing no target DNA, and "Method Blanks" are ultrapure sterile water samples filtered and processed like regular samples. Both NTCs and Blanks should have Cq values > 40 cycles. The "nd" values are defined as "non-detects", where the amplification fluorescent intensity never reaches the cycle threshold (Ct), thus a Cq value cannot be determined. The "Environmental LLOQ" is the expected LLOQ if 1 liter water samples are extracted, eluted into 100 µL eluate, then 2 µL eluate used per qPCR reaction.

References for Supplementary Materials:

- Campbell A, Fleisher J, Sinigalliano CD, White J, Lopez JV 2015. Dynamics of marine bacterial community diversity of the coastal waters of the reefs, inlets, and wastewater outfalls of Southeast Florida. MicrobiologyOpen. 4:390-408. DOI: 10.1002/mbo3.245.

- Griffith JF, Layton BA, Boehm AB, Holden PA, Jay JA, Hagedorn C, McGee CD, Weisberg SB. 2013. *The California Microbial Source Identification Manual: A Tiered Approach to Identifying Fecal Pollution Sources to Beaches.* Southern California Coastal Water Research Project (SCCWRP), Technical Report 804, 83 pp. Available at https://www.waterboards.ca.gov/water_issues/programs/beaches/cbi_projects/docs/sipp_manual.pdf (accessed 12 Feb 2020)

- Mieszkin S, Furet J-P, Corthier G, Gourmelon M. 2009. Estimation of Pig Fecal Contamination in a River Catchment by Real-Time PCR Using Two Pig-Specific Bacteroidales16S rRNA Genetic Markers. Appl. Environ. Microbiol. 75:3045-3054. DOI: 10.1128/AEM.02343-08

- Schriewer, A., C.D. Sinigalliano, K.D. Goodwin, A. Cox, D. Wanless, J. Bartkowiak, D.L. Ebentier, K.T. Hanley, J. Ervin, L.A. Deering, O.C. Shanks, L.A. Peed, W.G. Meijer, J. Griffith, J. Santo Domingo, J.A. Jay, P.A. Holden, and S. Wuertz. 2013. Performance evaluation of canine-associated Bacterioidales assays in a multi-laboratory comparison study. Water Research. 47:6909-6920. DOI: http://dx.doi.org/10.1016/j.watres.2013.03.062

- Shanks O, Atikovic E, Blackwood A, Lu J, Noble R, Domingo J, Seifring S, Sivaganesan M, Haugland R. 2008. Quantitative PCR for Detection and Enumeration of Genetic Markers of Bovine Fecal Pollution. *Appl. Environ. Microbiol.* 74:745-52. DOI: 10.1128/AEM.01843-07.

- Sinigalliano CD, Fleisher JM, Gidley ML, Solo-Gabriele HM, Shibata T, Plano LR, Elmir SM, Wanless D, Bartkowiak J, Boiteaua R, Withum K, Abdelzaher AM, He G, Ortega C, Zhub X, Wright ME, Kish J, Hollenbeck J, Backer LC, Fleming LE. 2010. Traditional and Molecular Analyses for Fecal Indicator Bacteria in Non-point Source Subtropical Recreational Marine Waters. Water Research. 44:3763-3772. DOI: 10.1016/j.watres.2010.04.026

- Sinigalliano CD, Ervin J, Van de Werfhorst JLC, Badgley B, Ballesté E, Bartkowiak J, Boehm AB, Byappanahalli M, Goodwin KD, Gourmelon M, Griffith J, Holden PA, Jay J, Layton B, Lee C, Lee J, Meijer WG, Noble R, Raith M, Ryu H, Sadowsky MJ, Schriewer

A, Wang D, Wanless D, Whitman R, Wuertz S, Santo Domingo JW. 2013. Multi-laboratory evaluations of the performance of *Catellicoccus marimammalium* PCR assays developed to target gull fecal sources. *Water Research* 47:6883–6896 DOI: 10.1016/j.watres.2013.02.059.

- Sinigalliano CD, Enochs IC, Stamates SJ, Jones JR, Featherstone CM, Gidley ML, Rosales SM, Gramer LJ, Staley C, and Carsey TP. 2019. Water quality and coral reef monitoring along the southeast Florida coast. NOAA Technical Report, OAR-AOML-47, 145 pp. DOI:10.25923/aanj-0912

- Staley C, Kaiser T, Gidley ML, Enochs IC, Jones PR, Goodwin KD, Sinigalliano CD, Sadowsky MJ, Chun CL. 2017. Differential impacts of land-based sources of pollution on the microbiota of southeast Florida coral reefs. *Applied and Environmental Microbiology* 83(10)e03378-16 DOI: 10.1128/aem.03378-16.

- Symonds EM, Sinigalliano CD, Gidley ML, Ahmed W, McQaig-Ulrich SM, and Breitbart M. 2016, Fecal pollution along the southeastern coast of Florida and insight into the use of pepper mild mottle virus as an indicator. *Journal of Applied Microbiology,* 121(5):1469-1481. DOI: 10.1111/jam.13252.

- US EPA. 2019. *Method 1696: Characterization of human fecal pollution in water by HF183/BacR287 Taqman quantitative polymerase chain reaction (qPCR) assay.* US Environmental Protection Agency, EPA-821-R-19-002. Washington, DC, 31 pp. Available at https://www.epa.gov/sites/production/files/2019-03/documents/method_1696_draft_2019.pdf (accessed 07 Nov 2020).
